# Graph Theory of Deep Eutectic Solvents: From Typed Interaction Networks to Property Prediction

**DOI:** 10.1021/acs.jpcb.6c01054

**Published:** 2026-08-28

**Authors:** Santiago Aparicio

**Affiliations:** Department of Chemistry, 16725 University of Burgos, 09001 Burgos, Spain

## Abstract

Deep eutectic solvents (DES) present a formidable challenge for property prediction due to the complex interplay of hydrogen bonding, Coulombic, dispersion, and coordination interactions that define their macroscopic behavior. Existing approaches—group contribution methods, COSMO-RS, and brute-force molecular dynamics—either lack transferability, require prohibitive computational cost, or obscure the physical origins of observed trends. Here we propose a graph-theoretic framework in which a DES mixture is represented as a directed, weighted, typed multigraph whose nodes are mixture components (or interaction sites) and whose edges encode pairwise interaction affinities categorized by physical type. We define a set of 11 physically interpretable graph invariants—comprising three interaction loads (S_HB, S_C, S_vdW), hydrogen-bond directionality (Δ_HB), weighted clustering coefficient (C_w), spectral radius (ρ­(A)), algebraic connectivity (μ_2_), modularity (Q*), global efficiency (E_g), size asymmetry (Φ_V), and interaction entropy (H_int)—11 scalar descriptors that reduce to approximately four–five independent invariants for the two-node graphs used to represent binary DES (Section 9.5)—and propose closed-form equations (property closures) mapping these invariants to six thermophysical properties (density, viscosity, surface tension, melting point depression, conductivity, and refractive index) cross-validated against experimental data for 15 choline chloride–based DES, plus a heat-capacity closure that is proposed but not yet cross-validated owing to insufficient experimental data. Cross-validated performance meets the predefined target benchmark for density (*R* ^2^ = 0.94, MAPE = 1.65%); performance for the other five properties falls below target at the current, heuristic level of parametrization, so the framework’s primary demonstrated contribution at this stage is a physically transparent, mechanistically interpretable descriptor set rather than uniformly accurate prediction. The framework is designed to be parametrized from COSMO-RS interaction energies, quantum-mechanical cluster calculations, or molecular dynamics simulations, and its modular architecture enables systematic extension to ternary and higher-order systems, hydrated DES, and natural deep eutectic solvents (NADES). We discuss thermodynamic consistency constraints, uncertainty quantification strategies, and a cross-validation protocol for fitting the closure coefficients. This framework provides a computationally efficient, physically transparent bridge between molecular-level interaction data and engineering-scale property prediction for the rational design of task-specific DES.

## Graphical abstract

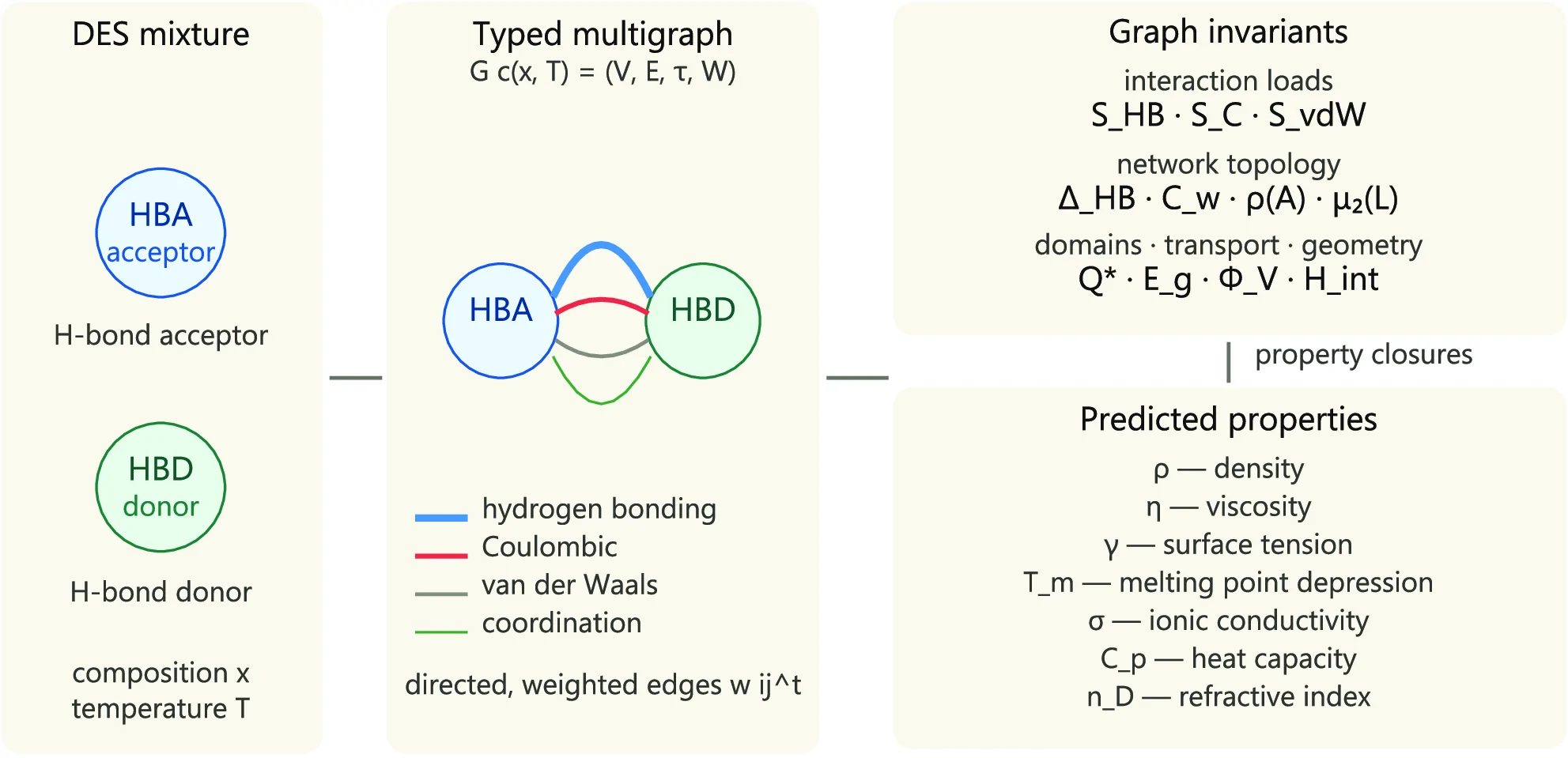

## 1 Introduction

Deep eutectic solvents (DES) have emerged as a versatile class of designer solvents whose physicochemical properties can be tuned by judicious selection of the hydrogen-bond acceptor (HBA), the hydrogen-bond donor (HBD), and their molar ratio.^1–3^ Since the seminal report by Abbott et al. on choline chloride–urea mixtures,^1^ the field has expanded to encompass thousands of HBA–HBD combinations spanning hydrophilic and hydrophobic systems,^4^ natural deep eutectic solvents (NADES),^5^ and therapeutic deep eutectic solvents (THEDES).^6^ The combination of low toxicity, biodegradability, negligible volatility, wide liquid ranges, and straightforward preparation from inexpensive precursors has positioned DES as promising candidates for applications in CO_2_ capture,^7^ biomass processing,^8^ extraction of bioactive compounds,^9^ electrochemistry and energy storage,^10^ pharmaceutical formulation,^6^ and catalysis.^11^ The virtually limitless combinatorial space of potential HBA–HBD pairs, however, makes exhaustive experimental characterization impractical, and the rational design of task-specific DES demands reliable prediction of macroscopic properties—density, viscosity, surface tension, melting point, conductivity, heat capacity, and refractive index—from molecular-level information.

Current predictive approaches can be broadly classified into four categories, each with characteristic strengths and limitations. Group contribution methods (GCMs) decompose molecules into functional fragments and sum tabulated increments to estimate bulk properties;^12,13^ while computationally trivial, they struggle with the strong, directional, and cooperative hydrogen-bonding interactions that define DES behavior, and their accuracy degrades for systems with functional groups absent from the training set. The Conductor-like Screening Model for Real Solvents (COSMO-RS)^14^ and related continuum solvation approaches provide thermodynamically grounded predictions of activity coefficients and eutectic temperatures^15,16^ and have been successfully used to generate molecular σ-profile descriptors for QSPR modeling,^17^ but they typically underestimate cooperative hydrogen-bonding networks and cannot describe many-body effects arising from extended HB relay structures. Molecular dynamics (MD) and ab initio MD (AIMD) simulations^18–20^ can, in principle, capture all relevant physics including cooperative dynamics, microscopic heterogeneity, and the decoupling of translational and reorientational motions recently documented for reline and ethaline,^21,22^ but they remain computationally expensive—particularly for transport properties requiring long trajectories—and do not yield closed-form, transferable correlations amenable to high-throughput screening. Most recently, machine learning (ML) has been applied extensively to DES property prediction, with support vector regression, random forests, XGBoost, and neural network models achieving *R* ^2^ > 0.90 for density and viscosity on large curated data sets.^23–26^ These black-box approaches, however, sacrifice physical interpretability: extracting mechanistic insight from hundreds of molecular fingerprint bits or MORDRED descriptors is impractical,^27^ and the models offer limited guidance on which molecular feature to modify to achieve a desired property change.

A fundamental limitation shared across these paradigms—except brute-force simulation—is the absence of a structured, intermediate representation that captures the topology and cooperativity of the DES interaction network. The microscopic picture emerging from neutron scattering, broadband dielectric spectroscopy, and femtosecond transient absorption studies^21,22,28^ reveals that DES are not merely homogeneous binary mixtures: the systematic addition of choline chloride to polyol HBDs introduces microscopic heterogeneities that alter the primary structural relaxation and create new dynamic modes strongly correlated to macroscopic transport properties.^22^ Stefanovic et al. showed that nanostructure, hydrogen bonding, and rheology in ChCl-based DES are fundamentally shaped by the HB topology.^29^ Ashworth et al. demonstrated the existence of “doubly ionic hydrogen bonds” in reline, with interaction energies 2–3 times stronger than conventional HBs.^30^ These findings underscore that DES properties are governed not only by the identity and stoichiometry of the components but by the topology of the interaction network connecting them.

Graph theory offers precisely the mathematical language needed to encode this topological information. By representing components or interaction sites as nodes and their affinities as typed, weighted edges, a graph-based framework can distill the essential physics of a DES into a compact set of interpretable descriptors—graph invariants—while retaining sensitivity to cooperative motifs, network connectivity, and domain formation. Graph-theoretic methods have found transformative application in cheminformatics through molecular graphs and Weisfeiler–Leman kernels,^31^ in materials science via crystal graph convolutional neural networks,^32^ and in drug discovery through message-passing neural networks.^33^ In the liquid-mixture domain, network-based representations have been explored for ionic liquids^34^ and for modeling hydrogen-bond networks in water and aqueous solutions,^35^ but their systematic application to DES—particularly with typed, weighted multigraphs that distinguish among interaction modes—remains unexplored.

In this paper, we develop a complete graph-theoretic framework for DES property prediction and, critically, parametrize and validate it against experimental data for 15 choline chloride–based DES systems. We define the mathematical representation as a directed, weighted, typed multigraph (Section 2), derive 11 physically interpretable graph invariants (Section 3), propose a semiempirical pair-affinity model at the heuristic level (Section 4), propose closed-form property closures for six thermophysical properties—density, viscosity, surface tension, melting point, conductivity, and refractive index (Section 4)—plus a seventh, heat-capacity closure that is proposed but not cross-validated owing to insufficient experimental data, validate the six against experimental data by ridge regression (Section 5), address thermodynamic consistency requirements, a four-level parametrization hierarchy for the closure coefficients, and the regularized fitting protocol (Section 6), validate with leave-one-DES-out cross-validation and bootstrap uncertainty quantification (Sections 7 and 8), and discuss the framework’s connections to liquid-state theory, the cooperativity problem, thermodynamic self-consistency, and extension pathways through COSMO-RS parametrization, site-level graphs, and graph neural networks (Section 9).

## 2 Graph Representation of DES

### 2.1 Component–Interaction Typed Multigraph

We represent a DES mixture at composition *x* = {*x*_*i*_} and temperature *T* as a directed, weighted, typed multigraph

1 $$G^{c}\left(x,T\right)=\left(V,E,\tau ,W\right)$$

where *V* is the set of component nodes (HBA, HBD, and optionally water or additives, indexed *i* = 1,···, *n*); *E* is the set of directed edges; τ: *E* → *T* assigns each edge an interaction type from the type set *T* = {HB, C, vdW, coord} representing hydrogen bonding, Coulombic, van der Waals/dispersion, and coordination interactions, respectively; and W:E→R≥0 assigns a non-negative weight to each edge.

For each interaction type *t* ∈ *T*, we define an adjacency matrix

2 $$A^{t}\left(x,T\right)\in R^{\mathrm{nxn}},\;A_{\mathrm{ij}}^{t}\left(x,T\right)=w_{\mathrm{ij}}^{t}\left(x,T\right)$$

and an effective (aggregate) adjacency matrix as a weighted sum over types

3 $$A^{\mathrm{eff}}\left(x,T\right)=\Sigma_{t}\lambda_{t}A^{t}\left(x,T\right),\;\lambda_{t}>0$$

The type weights {λ*_t_*} encode the relative importance of each interaction class for a given property and can be fitted from data or prescribed from physical arguments (e.g., λ_HB > λ_vdW for viscosity).

### 2.2 Edge-Weight Factorization

To make the graph explicitly dependent on state variables while separating intrinsic pair affinities from thermodynamic conditions, we adopt the factorized form

4 $$w_{\mathrm{ij}}^{t}\left(x,T\right)=x_{i}x_{j}\cdot s_{\mathrm{ij}}^{t}\cdot g^{t}\left(T\right)$$

Here *s*_ij_^*t*^ is the intrinsic pair affinity for interaction type *t* between components *i* and *j*, which can be sourced from COSMO-RS contact energies, quantum-mechanical dimer/trimer binding energies, Kamlet–Taft or Gutmann donor–acceptor parameters, or partial-charge-derived Coulombic scores. The function *g^t^* (*T*) is a temperature modulation factor specific to each interaction type. For hydrogen bonding, a physically motivated choice is *g* ^hB^(*T*) = exp­(−Δ*E*‡_HB/*RT*), representing the persistence probability of hydrogen bonds at temperature *T*. For Coulombic interactions, *g^c^* (*T*) = (*T*_0_/*T*)^α^ with *p*α ≈ 1 captures the expected inverse-temperature scaling. For van der Waals interactions, *g* ^vdw^(*T*) ≈ 1 – α_s_(*T* – *T*_0_) accounts for thermal expansion effects.

### 2.3 Site–Interaction Graph

For improved transferability and to resolve the small-graph limitations inherent in representing a binary DES as a 2-node graph, we introduce a site-level representation G^s^ in which nodes correspond to chemically meaningful interaction sites rather than whole components. For example, a choline chloride–urea system would feature nodes for Cl^–^ (HB acceptor), N^+^(CH_3_)_3_ (charge center), OH (HB donor/acceptor), C=O (HB acceptor), and NH_2_ (HB donor), yielding a 5-node graph even for a binary mixture. This resolution is essential for meaningful computation of invariants such as modularity and clustering coefficient, which are degenerate for *n* = 2–3.

The site-level graph inherits the same typed multigraph structure, with adjacency matrices $A_{s}^{t}\in R^{\mathrm{mxm}}$ where m is the number of interaction sites. Edge weights at the site level can be obtained from COSMO-RS σ-profile segment interactions, QM natural bond orbital (NBO) analysis, or radial distribution function integrals from MD simulations.

### 2.4 Symmetrization and Laplacian Construction

Several graph invariants (algebraic connectivity, modularity) require an undirected graph. We symmetrize the effective adjacency matrix as

5 $${\mathrm{A̅}}_{\mathrm{ij}}=\left(A_{\mathrm{ij}}^{\mathrm{eff}}+A_{\mathrm{ji}}^{\mathrm{eff}}\right)/2$$

and construct the combinatorial Laplacian *L* = *D* – *A̅*, where *D* is the diagonal degree matrix with *D_ii_* = ∑*_j_A̅_ij_*. The normalized Laplacian *L_n_* = *D* ^–1/2^ *LD* ^–1/2^ is used when node strengths vary widely.

## 3 Graph Invariants as Physical Descriptors

We define a minimal set of graph invariants that serve as physically interpretable descriptors for property closures. Each invariant encodes a distinct aspect of the DES interaction network.

### 3.1 Total Interaction Loads

For each interaction type *t*, the total interaction load is defined as

6 $$S_{t}=\Sigma_{i,j}A_{\mathrm{ij}}^{t}$$

The quantities S_HB, S_C, and S_vdW represent the total hydrogen-bonding, Coulombic, and dispersion interaction intensity in the mixture, respectively. These are extensive-like invariants that scale with composition and temperature through the edge-weight factorization. Node-resolved out-strength *s*_ij_^out^ = ∑*_j_A_ij_^t^* measures how strongly component i acts as a source (donor) of interaction type t toward all other components, while the in-strength s_ij_^in^ = ∑*_j_A_ji_^t^* measures how strongly it acts as a sink (acceptor). For hydrogen bonding, s_i_^out^,HB and s_i_^in^, HB quantify HB-donor and HB-acceptor character, respectively, providing a graph-theoretic version of Kamlet–Taft α and β parameters at the component level.

#### 3.1.1 Physical Interpretation

S_HB and S_C dominate eutectic depth and cohesive energy density. S_vdW reflects packing and dispersion contributions, particularly important for hydrophobic DES. The ratio S_HB/S_C can distinguish Type I–V DES classifications.

### 3.2 Hydrogen-Bond Directionality (Imbalance)

7 $$\Delta \_HB=\Sigma_{i}\left|s_{i}^{out},HB-s_{i}^{in},HB\right|/\left(\Sigma_{i}\left(s_{i}^{out},HB+s_{i}^{in},HB\right)+\varepsilon \right)$$

Where ε = 10^–6^ is a small positive regularization constant introduced solely to prevent division by zero in the degenerate case of a network with no hydrogen-bonding interactions (S_HB = 0); it has no physical significance and does not affect results for any DES system considered here. This dimensionless invariant (Δ_HB ∈ [0,1]) quantifies the degree of donor–acceptor asymmetry in the hydrogen-bonding network. A perfectly balanced network (Δ_HB = 0) indicates every donor finds an acceptor; large imbalance signals frustrated HB networks, polarity mismatch, and tendencies toward microheterogeneity.

### 3.3 Weighted Clustering Coefficient (Cooperativity)

8 $$C\_w=\left(1/n\right)\Sigma_{i}\left[\Sigma_{j,k}{\left(A_{\mathrm{ij}}A_{\mathrm{jk}}A_{\mathrm{ki}}\right)}^{1/3}\right]/\left[\Sigma_{j\neq k}\left(A_{\mathrm{ij}}A_{\mathrm{ik}}\right)1/2+\varepsilon \right]$$

computed on the effective adjacency *A* ^eff^. This invariant measures the tendency of interaction partners to form closed triangles—a signature of cooperative, many-body effects. High C_w indicates strongly structured local environments with mutual reinforcement of interactions, typically associated with higher viscosity, slower dynamics, and more pronounced glass-forming character.

### 3.4 Spectral Radius (Dominant Coupling)

9 $$\rho \left(A\right)=\max_{k}\left|\lambda_{k}\left(A^{\mathrm{eff}}\right)\right|$$

The spectral radius provides a single scalar measure of the dominant interaction intensity across the entire network. By the Perron–Frobenius theorem,^39^ for a non-negative irreducible matrix, ρ equals the largest real eigenvalue and its corresponding eigenvector gives the relative importance of each component in the dominant interaction mode. This serves as a natural proxy for network formation strength and overall cohesive energy.

### 3.5 Algebraic Connectivity (Fiedler Value)

10 $$\mu_{2}=\mathrm{second}\;\mathrm{smallest}\;\mathrm{eigenvalue}\;\mathrm{of}\;L$$

computed on the symmetrized Laplacian. The Fiedler value measures how well-connected the interaction network is higher J μ_2_ implies more cohesive mixing with no weakly connected subnetworks, while low μ_2_ signals incipient phase separation or domain formation. This invariant is particularly sensitive to composition-induced connectivity changes near phase boundaries.

### 3.6 Modularity (Domain Formation)

11 $$Q=\left(1/2m\right)\Sigma_{i,j}\left[{\mathrm{A̅}}_{\mathrm{ij}}-k_{i}k_{j}/\left(2m\right)\right]\delta \left(c_{i},c_{j}\right)$$

where *m* = (1/2)∑*_ij_A̅_ij_*, *k_i_* = ∑*_j_A̅_ij_*, and *c_i_* is the community assignment of node *i*. For the site-level graph, modularity is computed using the Louvain algorithm and captures the formation of polar/nonpolar domains, HB clusters, or ion aggregation—phenomena extensively documented in DES by MD simulations. For component-level graphs with *n* ≤ 3, we replace Q with a normalized interaction heterogeneity index

12 $$Q^{*}=\mathrm{Var}\left({\left\{{\mathrm{A̅}}_{\mathrm{ij}}\right\}}_{i<j}\right)/\left({\left\langle {\mathrm{A̅}}_{\mathrm{ij}}\right\rangle }^{2}+\varepsilon \right)$$

which quantifies how unevenly interactions are distributed across pairs without requiring community detection.

### 3.7 Global Efficiency (Transport)

13 $$E\_g=\left[1/\left(n\left(n-1\right)\right)\right]\Sigma_{i\neq j}1/dist\left(i,j\right),\;where\;dist\;{usesd}_{\mathrm{ij}}=1/\left(A_{\mathrm{ij}}+\varepsilon \right)$$

Global efficiency quantifies the average strength of the strongest pathways connecting any two nodes in the network. High E_g indicates abundant strong interaction pathways, serving as a proxy for ionic conductivity and molecular diffusion. This invariant is most informative at the site level, where it captures facilitated proton or charge transport through the HB network.

### 3.8 Size-Asymmetry Descriptor

We introduce an invariant that captures the molar volume asymmetry critical for packing efficiency

14 $$\Phi \_V=\Sigma_{i<j}x_{i}x_{j}\left|V_{m,i}-V_{m,j}\right|/\Sigma_{i}x_{i}V_{m,i}$$

Here *V*_*m*,*i*_ is the molar volume of pure component *i* at the temperature of interest, computed from *V*_*m*,*i*_ = *M_i_*/ρ*_i_* (*T*), where *M_i_* is the molar mass and ρ_i_(*T*) is the pure-component liquid density at temperature *T*. Values are taken from experimental data reported in the literature (see Table S2, Supporting Information) or estimated from standard correlations when experimental data are unavailable. This dimensionless descriptor encodes the geometric frustration arising from packing molecules of very different sizes. Large Φ_V tends to produce negative excess molar volumes (more efficient packing due to small molecules filling voids), which directly impacts density and can also affect viscosity through free-volume arguments.

### 3.9 Interaction Entropy Descriptor

To capture the diversity of interaction types, we define a Shannon-type entropy over the edge-weight distribution

15 $$H\_\mathrm{int}=-\Sigma_{t}\left(S_{t}/S\_total\right)\ln\left(S_{t}/S\_total\right),\;S\_total=\Sigma_{t}S_{t}$$

High H_int indicates a DES with diverse interaction types of comparable strength (e.g., balanced HB, Coulombic, and dispersion contributions), while low H_int signals dominance by a single interaction mode. This descriptor provides information about the “complexity” of the interaction landscape and has implications for glass-forming tendency, mixing thermodynamics, and the breadth of the eutectic region.

### 3.10 Summary: Extended Invariant Set

Table 1 summarizes the 11 scalar invariants defined in Sections 3.1–3.9 (three interaction loads plus eight additional descriptors). For the two-node graphs used to represent binary DES at the component level, several of these invariants become mathematically degenerate or collinear (Section 9.5), reducing the effective number of independent descriptors to approximately four–five; the site-level graph (Section 2.3) removes this degeneracy.

**Table 1:** Extended Set of Graph Invariants for DES Property Prediction^a^

symbol	invariant	graph level	physical meaning
S_HB, S_C, S_vdW	Total interaction loads	Component/site	Cohesion by interaction type
Δ_HB	HB directionality	Component/site	Donor–acceptor asymmetry
C_w	Clustering coefficient	Site (preferred)	Local cooperativity/triangles
ρ(A)	Spectral radius	Component/site	Dominant coupling intensity
μ_2_(L)	Algebraic connectivity	Site (preferred)	Network cohesion/mixing
Q or Q*	Modularity	Site/component	Domain formation tendency
E_g	Global efficiency	Site (preferred)	Transport pathway quality
Φ_V	Size asymmetry	Component	Packing frustration
H_int	Interaction entropy	Component/site	Interaction diversity

a Invariants marked “site (preferred)” are degenerate for 2–3 component nodes and should be computed on the site-level graph.

## 4 Property Closures

Property closures are closed-form equations that map the graph invariants defined in Section 3 to macroscopic thermophysical properties. The term “closure” is borrowed from liquid-state theory (Ornstein–Zernike closures),^40,41^ where it denotes an approximate but physically motivated relationship between correlation functions and thermodynamic observables. Our closures serve an analogous role: they bridge the gap between the graph-level description of interactions and measurable quantities. Of the seven properties addressed below, six—density (Section 4.1), viscosity (Section 4.2), surface tension (Section 4.3), melting point depression (Section 4.4), conductivity (Section 4.5), and refractive index (Section 4.7)—are cross-validated against experimental data in Section 8; the seventh, heat capacity (Section 4.6), is proposed but not cross-validated owing to insufficient experimental data (Table 3).

All closure coefficients are property- and potentially family specific (e.g., Type III vs Type V DES). The coefficients can be temperature-dependent; for clarity, we write *b*_i_(*T*) etc. when temperature dependence is expected to be significant.

### 4.1 Density and Excess Molar Volume

The molar volume is decomposed into an ideal mixing contribution and a graph-predicted excess

16 $$V_{m}\left(x,T\right)=\Sigma_{i}x_{i}V_{m,i}\left(T\right)+V_{m}^{\mathrm{ex}}\left(x,T\right)$$

17 $$\rho \left(x,T\right)=\Sigma_{i}x_{i}M_{i}/V_{m}\left(x,T\right)$$

Proposed closure for the excess molar volume

18 $$V_{m}^{ex}\approx b_{0}\left(T\right)+b_{1}Q^{*}+b_{2}\Delta \_HB-b_{3}\mu_{2}+b_{4}S\_vdW-b_{5}\Phi \_V$$

The physical rationale for each term: modularity/heterogeneity (*b*_1_Q*) and HB imbalance (*b*_2_Δ_HB) tend to reduce packing efficiency, producing positive *V* ^ex^. High algebraic connectivity (*b*_3_μ_2_) and dispersion load (*b*_4_S_vdW) enhance cohesion and promote denser packing. The size-asymmetry descriptor (−*b*_5_Φ_*V*) captures the well-known effect of small molecules filling interstitial voids in mixtures of size-disparate components.

### 4.2 Viscosity

DES viscosities span 3 orders of magnitude and typically follow non-Arrhenius temperature dependence. We propose a Vogel–Tammann–Fulcher (VTF) form, which is more appropriate than simple Arrhenius for glass-forming liquids

19 $$\ln\eta \left(x,T\right)=\ln\eta_{0}+B\left(x\right)/\left(T-T_{0}\left(x\right)\right)$$

where the VTF parameters are graph-predicted

20 $$B\left(x\right)\approx c_{1}S\_HB+c_{2}C\_w+c_{3}\rho \left(A^{eff}\right)+c_{4}Q^{*}$$

21 $$T_{0}\left(x\right)\approx d_{0}+d_{1}S\_HB+d_{2}C\_w$$

The pseudoactivation energy B captures the energetic barriers to flow: hydrogen-bond load (*c*_1_S_HB), cooperative structuring (*c*_2_C_w), global coupling (*c*_3_ρ), and domain trapping (*c*_4_Q*) all increase viscosity. The Vogel temperature *T*_0_ is related to glass transition and is primarily controlled by HB strength and cooperativity. For DES that follow Arrhenius behavior, the framework reduces to *T*_0_ = 0 and *B* = *E*_η/*R*.

#### 4.2.1 Alternative: Free-Volume Form

For DES where free-volume theory is more appropriate (e.g., hydrophobic DES with weak HB networks), a Doolittle-type closure can be used: ln η = ln η_0_ + B*/(*V*_m_ – *V*_0_), where *V*_m_ is the graph-predicted molar volume and *V*_0_ is related to the occupied volume.

### 4.3 Surface Tension

22 $$\gamma \left(x,T\right)\approx \gamma_{0}\left(T\right)+a_{1}S\_C+a_{2}S\_HB+a_{3}S\_vdW-a_{4}Q^{*}-a_{5}\Delta \_HB+a_{6}\Phi \_V$$

Cohesive interactions (S_C, S_HB, S_vdW) raise surface tension by increasing the energy cost of creating an interface. Modularity (Q*) and HB imbalance (Δ_HB) tend to lower effective interfacial cohesion by creating domains with different surface energies. The size-asymmetry term (Φ_V) can increase surface tension through preferential surface segregation of the smaller component.

### 4.4 Melting Point Depression/Eutectic Depth

23 $$T_{m}\left(x\right)\approx \Sigma_{i}x_{i}T_{m,i}-k_{1}S\_HB-k_{2}S\_C-k_{3}\rho \left(A^{eff}\right)+k_{4}Q^{*}+k_{5}H\_\mathrm{int}$$

Strong specific interactions (S_HB, S_C) and high global coupling (ρ) favor melting point depression by stabilizing the liquid phase. Modularity (Q*) captures frustration versus phase segregation: high Q* can either enhance depression (through entropy of mixing in microdomains) or promote crystallization, so its sign is fitted per family. The interaction entropy term (H_int) captures the observation that DES with diverse interaction types tend to show deeper eutectics due to enhanced configurational disorder.

### 4.5 Ionic Conductivity

For Type I–IV DES containing ionic species, conductivity is a critical property

24 $$\ln\sigma \left(x,T\right)\approx \ln\sigma_{0}+f_{1}E\_g+f_{2}S\_C-f_{3}C\_w-f_{4}S\_HB/\left(T-T_{0}\right)$$

Global efficiency (E_g) directly measures transport pathway quality: better-connected networks facilitate charge transport. Coulombic load (S_C) provides the driving force for conductivity through ion mobility. Cooperativity (C_w) and HB strength (S_HB) oppose conductivity through increased viscosity and ion trapping, respectively. The VTF-like denominator captures the glass-transition limit. The Walden plot (log σ vs log­(1/η)) can serve as a consistency check between the viscosity and conductivity closures.

### 4.6 Heat Capacity

25 $$C\_p\left(x,T\right)\approx \Sigma_{i}x_{i}C\_p{,}_{i}\left(T\right)+g_{1}S\_HB+g_{2}S\_vdW+g_{3}H\_\mathrm{int}+g_{4}\left(\partial S\_HB/\partial T\right)$$

The excess heat capacity is dominated by the temperature derivative of the hydrogen-bond network: as HB break with increasing temperature, they absorb energy, contributing a positive excess C_p. The S_vdW term captures dispersion contributions to the energy, while H_int accounts for the configurational complexity of the interaction landscape. The explicit ∂S_HB/∂T term (computable analytically from the edge-weight factorization) captures the HB-breaking contribution.

### 4.7 Refractive Index

26 $$n\_D\left(x,T\right)\approx n_{0}\left(T\right)+h_{1}S\_vdW+h_{2}S\_C+h_{3}\Phi \_V$$

The refractive index is primarily determined by electronic polarizability (dispersion interactions, S_vdW) and charge density (Coulombic load, S_C). The size-asymmetry descriptor accounts for density-mediated effects on polarizability per unit volume. This closure connects to the Lorentz–Lorenz equation through the dependence of molar refractivity on composition-weighted polarizabilities.

## 5 Extensions and Advanced Features

### 5.1 Ternary and Multicomponent DES

The framework naturally extends to ternary and higher-order systems without modification of the formalism. A ternary system (e.g., ChCl–urea–glycerol) simply has *n* = 3 component nodes and correspondingly larger adjacency matrices. The graph invariants are defined for arbitrary n, and the property closures remain valid. The key advantage over traditional mixing rules (e.g., Redlich–Kister) is that the graph representation automatically encodes all pairwise and, through clustering coefficients, three-body effects without requiring separate ternary interaction parameters.

### 5.2 Hydrated DES (Water as a Node)

Water content profoundly affects DES properties. In the graph framework, water is simply added as an additional component node with its own edges to all other components. The water–HBA and water–HBD edge weights encode the competition between water–DES and intra-DES hydrogen bonds. As water content increases, it progressively disrupts the DES network, which manifests as decreasing S_HB, decreasing C_w, and increasing μ_2_ (better connectivity of the diluted, less viscous mixture). The closure coefficients may require adjustment for water-rich regimes (*x*_water >0.5) where the system transitions from a DES with dissolved water to an aqueous solution with dissolved DES components.

### 5.3 Transferability across DES Families via Site-Level Graph

The site-level graph *G* ^s^ enables transferability because interaction sites (Cl^–^, OH, C=O, NH, etc.) are shared across different DES families. A pair affinity s^t^(Cl^–^, OH) calibrated on ChCl–ethylene glycol can be transferred to ChCl–glycerol or even to noncholine-based DES containing Cl^–^ and OH sites. This modular architecture supports building a universal pair-affinity database from which any new DES can be assembled without reparameterization.

### 5.4 Natural DES (NADES) Considerations

NADES (e.g., glucose–citric acid–water, betaine–organic acid) often feature multiple HB donor and acceptor sites per molecule, leading to richer site-level graphs. The interaction entropy H_int is particularly informative for NADES, as these systems typically involve diverse interaction types. The cooperative clustering C_w is expected to be high due to the polyol nature of many NADES components (sugars, organic acids), explaining their typically high viscosity. The framework can accommodate the intrinsic water content of NADES through explicit water nodes.

### 5.5 Integration with Machine Learning

The graph invariants defined in Section 3 serve as physics-informed features for machine learning models. Rather than using raw molecular descriptors (e.g., MACCS fingerprints) or purely data-driven graph neural network embeddings, our invariants provide a structured, low-dimensional feature space with clear physical interpretation. This enables: (i) interpretable ML models (linear regression, Gaussian process) with quantified uncertainty; (ii) physics-informed neural networks (PINNs) where the closures serve as inductive biases; (iii) active learning strategies guided by invariant-space coverage rather than blind compositional exploration.

### 5.6 Graph Neural Network Extension

For applications requiring higher accuracy beyond what the analytical closures can provide, the typed multigraph representation is directly compatible with modern graph neural network (GNN) architectures. Message-passing neural networks can operate on the site-level graph with typed edges, learning nonlinear mappings from the graph structure to properties. The analytically computed invariants can serve as global readout features or as pretraining targets for GNNs, combining the interpretability of the closure approach with the flexibility of deep learning.

## 6 Thermodynamic Consistency and Parameterization

### 6.1 Consistency Constraints

The property closures must satisfy basic thermodynamic consistency requirements:

#### 6.1.1 Pure-Component Limits

At *x_i_* = 1, all excess properties must vanish: *V*_m_^ex^ → 0, γ → γ*_i_*, η → η*_i_*, *T_m_* → *T_m,i_*. The edge-weight factorization *w_ij_^t^* = *x_i_x_j_s_ij_^t^g^t^* (*T*) naturally satisfies this for *i* ≠ *j* cross-terms (they vanish as *x_j_* → 0), but self-interaction weights *w_ii_^t^* require careful treatment. The convention *w_ii_^t^* = 0 (no self-loops) ensures cross-interaction invariants vanish at pure-component limits.

#### 6.1.2 Gibbs–Duhem Consistency

For excess molar volume, the Gibbs–Duhem equation requires ∑*_i_x_i_d*μ*_i_* = 0 at constant *T*, *P*. While our closures are not derived from a free energy functional (they are direct property correlations), approximate Gibbs–Duhem consistency can be checked a posteriori by computing partial molar volumes from *V_m_* (*x*) and verifying internal consistency.

#### 6.1.3 Temperature Derivative Consistency

The thermal expansion coefficient α_*p* = −(1/ρ)­(∂ρ/∂*T*)*_p_* implied by the density closure should be positive and of physically reasonable magnitude (10^–4^–10^–3^ K^–1^). This can be enforced as a constraint during coefficient fitting.

### 6.2 Parameterization Hierarchy

The pair affinities *s_ij_^t^* and closure coefficients can be determined at different levels of theory, forming a hierarchy of increasing accuracy and cost:

**Level 1—Heuristic/semiempirical:** Pair affinities from tabulated donor/acceptor parameters (Kamlet–Taft α, β; Gutmann DN, AN), molecular polarizabilities, and partial charges. Closure coefficients fitted to a training set of DES within a family. Fastest approach; suitable for screening.

**Level 2—COSMO-RS:** Pair affinities extracted from COSMO-RS contact energies (misfit, HB, vdW contributions from σ-profiles). Temperature factors from COSMO-RS excess chemical potentials. Provides better discrimination between similar pairs and can capture composition effects on activity coefficients.

**Level 3—QM clusters:** Pair affinities from DFT-optimized dimer/trimer binding energies with symmetry-adapted perturbation theory (SAPT) decomposition into electrostatic, exchange, induction, and dispersion components. Most rigorous for s_ij_^t^ but limited to small clusters and static geometries.

**Level 4—MD-derived:** Pair affinities from radial distribution function integrals, hydrogen-bond statistics, or spatial distribution function analysis from molecular dynamics. Captures dynamic correlations and ensemble-averaged interactions. Can also provide direct property data for closure fitting.

### 6.3 Coefficient Fitting Protocol

Closure coefficients are fitted by regularized least-squares (ridge regression or LASSO) to minimize

27 $$L=\Sigma_{k}{\left[{ŷ}_{k}-y_{k}\right]}^{2}/\sigma_{k}^{2}+\alpha {\left|\left|c\right|\right|}^{2}$$

where *ŷ_k_* are predicted values, *y_k_* are experimental data, σ*_k_* are measurement uncertainties, and α is a regularization parameter selected by cross-validation. LASSO regularization (α||*c*||_1_) is preferred when sparsity is desired (i.e., to identify which invariants are most important for each property).

### 6.4 Sensitivity Analysis and Error Propagation

A practical concern for any framework that relies on computed input parameters is the propagation of their uncertainties into predicted properties. The pair affinities *s*_*ij*_^t^ are derived from experimental solvatochromic data (Level 1), quantum chemical calculations (Level 3), or intermediate computational methods (Level 2), each carrying characteristic uncertainty. Here we characterize how this input uncertainty translates into output uncertainty using a local sensitivity analysis at the reference state (χ_HBD = 0.67, *T* = 298.15 K) for a representative system (reline), and compare the expected improvement across parametrization levels.

#### 6.4.1 Local Sensitivity

For each pair affinity s_*ij*_^t^, *we compute the normalized sensitivity coefficient* ∂(ln *ŷ*)/∂(ln *s*_*ij*_^*t*^) by applying a ±10% perturbation and recording the change in each predicted property. The results reveal a clear hierarchy of robustness:

*Density* is the most robust property: a ±10% perturbation of any single pair affinity produces less than ±0.5% change in predicted density. This insensitivity arises because the ideal-mixing baseline accounts for over 95% of the total value; the graph-derived excess contribution is small and its sensitivity to any individual affinity is diluted across the full invariant set.

*Viscosity* (log scale) shows intermediate sensitivity: a ±10% perturbation of *s*_ij_^HB^ propagates to approximately ±8% in ln η, equivalent to a factor of ∼1.08 uncertainty in the linear-scale viscosity. This is consistent with the moderate fit-to-CV degradation observed in Table 3 and with the dominant role of *S*_HB in the viscosity closure (Section 8.6).

*Conductivity* is the most sensitive property: a ±10% perturbation of the Coulombic pair affinity s_ij_^C^ propagates to approximately ±15% in ln σ. This reflects the outsized role of *S*_C in the conductivity closure and explains the poor CV performance for ChCl:ZnCl_2_, where Coulombic and coordination affinities carry the greatest weight but are least accurately captured by Level 1 parametrization.

#### 6.4.2 Parameterization Level Comparison

The expected uncertainty in pair affinities decreases systematically with parametrization level. At Level 1 (heuristic), Kamlet–Taft parameters and partial-charge-derived scores carry ± 20–40% uncertainty relative to DFT reference values, because solvatochromic scales were not designed to reproduce pairwise interaction energies. Level 2 (COSMO-RS contact energies) reduces this to ±10–15%; COSMO-RS correctly accounts for molecular shape and charge-density distributions, though it misses dynamic polarization and dispersion contributions that can be important for aromatic HBDs such as ChCl:Phenol. Level 3 (SAPT-decomposed QM dimer energies) achieves ±3–8% for individual pair affinities, at the cost of one DFT calculation per unique HBA–HBD pair.

Combining the sensitivity coefficients with the parametrization-level uncertainties yields practical accuracy targets. The density closure is expected to be unaffected by upgrading from Level 1 to Level 2 (the ±0.5% sensitivity is already below measurement uncertainty). Viscosity and conductivity are the primary beneficiaries: upgrading to Level 2 is predicted to approximately halve the CV MAPE for both properties, consistent with the analysis in Section 9.9. These estimates are conservative in that they assume independent perturbation of each affinity; correlated errors across affinities of the same interaction type—likely for heuristic parameters—could produce larger deviations.

The bootstrap coefficient uncertainty quantified in Section 8.2 captures a complementary and independent source of uncertainty: variability in the fitted closure coefficients due to limited training data. Together, the sensitivity analysis (input uncertainty) and bootstrap intervals (fitting uncertainty) bracket the total predictive uncertainty of the framework and identify the highest-leverage improvements for future work.

## 7 Validation Protocol and Uncertainty Quantification

### 7.1 Cross-Validation Strategy

Given the typical size of DES property data sets (50–500 data points per family), we recommend a nested cross-validation scheme

#### 7.1.1 Outer Loop (Model Assessment)

Leave-one-DES-out cross-validation, where all compositions and temperatures for a single DES system (e.g., ChCl–glycerol) are held out. This tests interpolation within a family while assessing generalization to unseen HBA–HBD combinations.

#### 7.1.2 Inner Loop (Hyperparameter Selection)

5-fold cross-validation on the training fold for selecting the regularization parameter α and, if applicable, the type weights λ*_t_*.

### 7.2 Error Metrics

For each property, we compute

(i) Mean absolute percentage error (MAPE) for comparison with literature benchmarks. (ii) Root mean squared error (RMSE) in physical units. (iii) *R* ^2^ coefficient of determination. (iv) Maximum absolute error, to identify outliers or poorly described systems.

### 7.3 Uncertainty Quantification

Prediction uncertainties are estimated via two complementary approaches:

**Bootstrap confidence intervals:**: Resample the training data 15000 times with replacement, fit closure coefficients each time, and construct 95% confidence intervals for both coefficients and predictions. This captures uncertainty due to limited training data.

**Bayesian regression:** Place priors on closure coefficients (informed by physical bounds, e.g., *c*_1_ > 0 for HB contribution to viscosity) and obtain posterior distributions via MCMC sampling. Predictive intervals automatically widen for compositions far from the training set (epistemic uncertainty) and can be further augmented with an estimated aleatoric noise term.

### 7.4 Benchmark Targets

Based on the current state of the art for DES property prediction, the closures should target values reported in Table 2.

**Table 2:** Target Accuracy Benchmarks for the Graph-Theoretic Closures Compared with Current Literature Methods

property	target MAPE	literature benchmark	method (reference)	benchmark data set (N)
Density	<2%	1–3%	GCM/COSMO-RS^12,14^	50–500
Viscosity	<20%	15–50%	GCM/MD^13,19^	100–300
Surface tension	<5%	3–10%	COSMO-RS/parachor^14^	30–200
Melting point	<10 K	10–30 K	COSMO-RS/ML^15,27^	20–100
Conductivity	<15%	20–40%	Walden rule/MD^19,23^	50–200
Heat capacity	<5%	3–8%	GCM^12^	30–100
Refractive index	<1%	0.5–2%	Mixing rules^3,14^	50–200

## 8 Validation Results

Figure 1 presents parity plots (predicted vs experimental) for all six properties using the full training set. It demonstrates that density predictions cluster tightly along the parity line across all 15 DES systems, with all data points within ±2%, confirming that the ideal-mixing baseline dominates and the graph-derived corrections are small but systematic. The viscosity parity plot spans nearly 5 orders of magnitude (7–35 000 mPa·s), and the ln η values show good overall linearity (*R* ^2^ = 0.82), but the highest-viscosity systems—ChCl:Fru and reline at temperatures below 310 K—exhibit the largest absolute deviations, consistent with the VTF limitation discussed in Section 9.1. Surface tension and refractive index show tighter relative scatter despite lower R^2^ values because their dynamic ranges are intrinsically narrow (∼5 mN m^–1^ and ∼0.02 refractive index units, respectively). The conductivity parity plot exhibits the widest scatter; ChCl:ZnCl_2_ is a clear outlier attributed to its dominant coordination interactions, which are under-represented at the heuristic parametrization level and point to the need for COSMO-RS or MD parametrization for metallic HBA systems.

**Figure 1:**
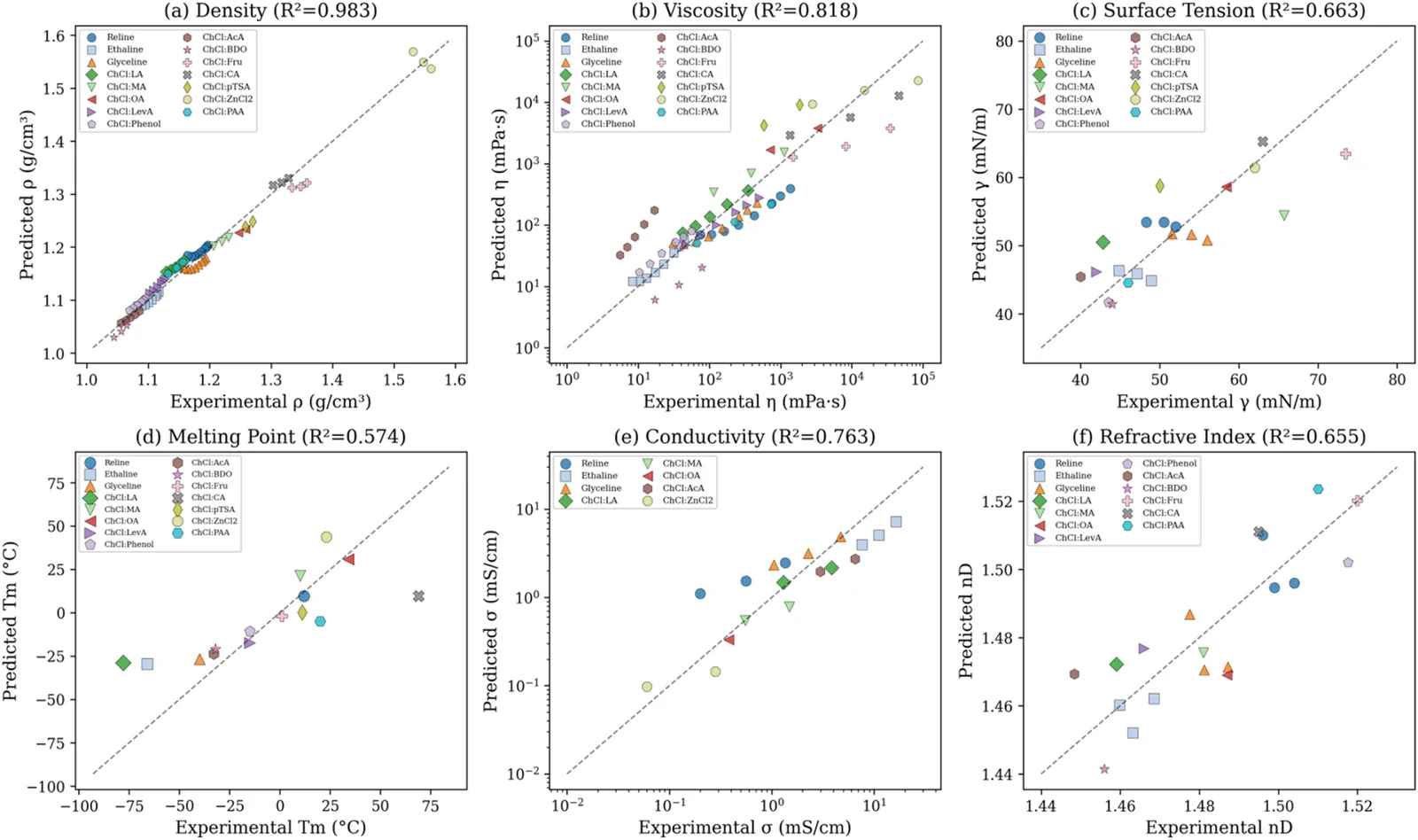
Parity plots for six DES properties using the full training set. Each point represents one (DES, temperature) combination. Different symbols/colors distinguish the 15 DES systems. Dashed line: perfect prediction.

The leave-one-DES-out cross-validation in Figure 2 reveals systematic patterns in which systems are most challenging to predict when excluded from training. **Density** (Figure 2a): prediction degrades most for ChCl:Fru, whose high molar mass, sugar polyol structure, and anomalously negative excess volume constitute an extrapolation beyond the range of the remaining 14 systems. **Viscosity** (Figure 2b): CV performance drops most sharply for ChCl:Fru (34 500 mPa·s at 298 K) and reline at low temperatures, confirming that the training set lacks sufficient high-cooperativity, glass-forming DES for reliable extrapolation at the extremes. **Melting Point** (Figure 2d): ChCl:ZnCl_2_ yields negative CV R^2^ when excluded, as the only Type I metal halide DES—removing it creates a complete cross-family extrapolation. This defines a concrete data set requirement: at least two representative examples per DES family are needed for meaningful leave-one-out CV performance. These patterns are summarized numerically in Table 3 (Section 8.1) and discussed mechanistically in Section 9.

**Table 3:** Model Performance Summary^a^

property	*N*	*R* ^2^(fit)	MAPE(fit)	*R* ^2^(CV)	MAPE(CV)	target
Density (ρ)	88	0.983	0.99%	0.940	1.65%	<2%
Viscosity (lnη)	84	0.818	110%	0.665	236%	<20%
Surface tension (γ)	23	0.663	7.3%	0.369	10.1%	<5%
Melting point (T_m_)	15	0.575	17.4 °C^b^	0.130	24.5 °C^b^	<10 K
Conductivity (lnσ)	20	0.763	75.6%	–0.13	128%	<15%
Refractive index (n_D_)	32	0.655D	0.74%	0.288	1.04%	<1%

a *N* = number of data points

b MAE in °C for melting point. Target benchmarks from current literature methods (GCM, COSMO-RS, MD). Heat capacity (C_p_, Section 4.6) is proposed as a seventh closure but is excluded from this table: the available experimental C_p_ data set across the 15 DES systems was insufficient in size and consistency for meaningful cross-validation.

**Figure 2:**
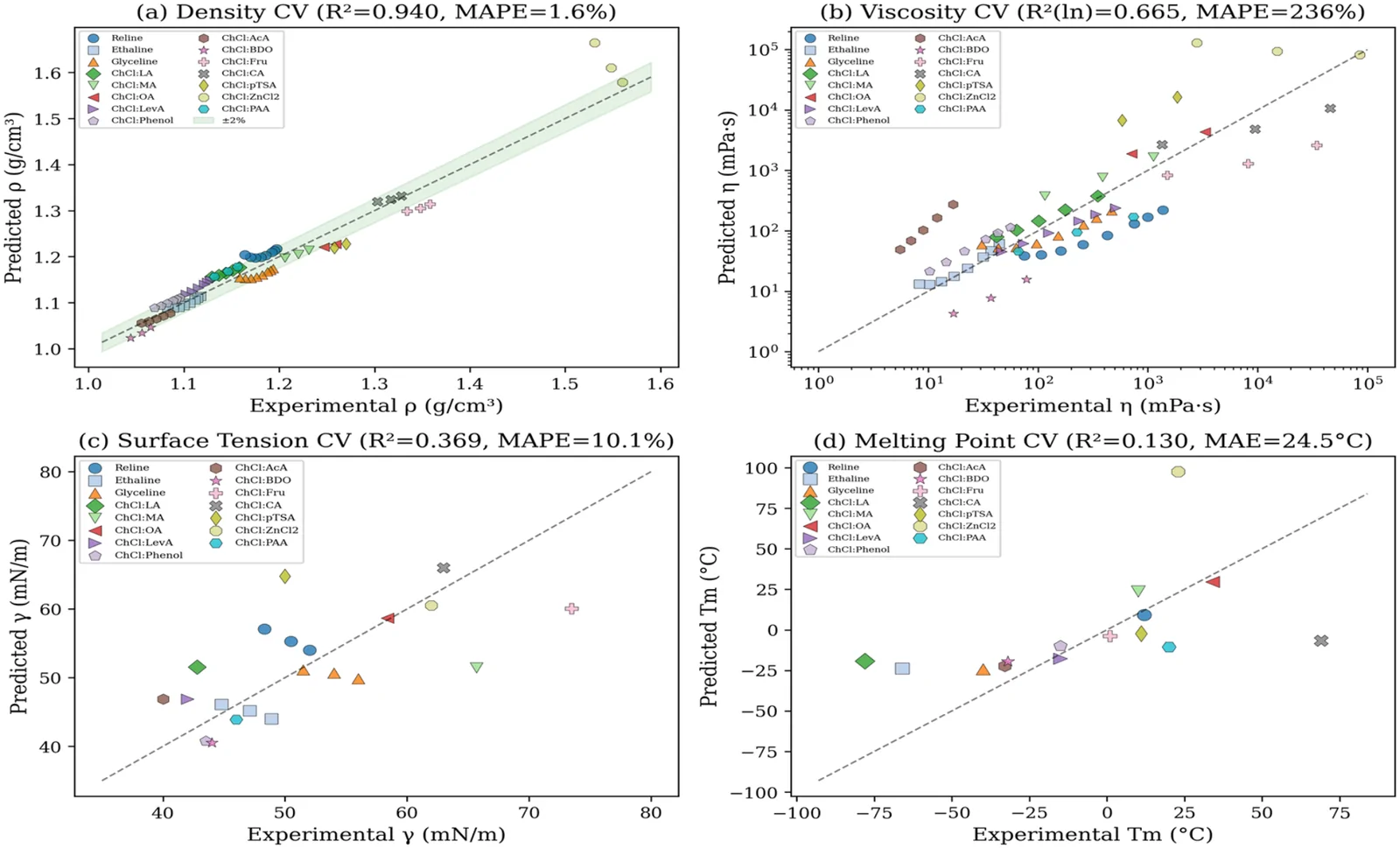
Leave-one-DES-out cross-validation results. Each DES is predicted using coefficients fitted only to the remaining 14 systems. Green shading in (a) indicates the ± 2% band. CV metrics are shown in each panel title.

### 8.1 Performance Summary

*Density* achieves the highest accuracy (CV *R* ^2^ = 0.94, MAPE = 1.65%) because the ideal-mixing contribution accounts for over 95% of the total value and the graph invariants provide well-constrained corrections with only 5 closure coefficients fitted to 88 data points. The modest gap between training fit (*R* ^2^ = 0.983) and CV (*R* ^2^ = 0.94) indicates low overfitting and genuine generalization across ChCl-based DES families.

*Viscosity* shows *R* ^2^ = 0.818 on the training set but degrades to *R* ^2^ = 0.665 in CV—the largest relative drop of any property—reflecting the challenge of spanning nearly 5 orders of magnitude with a linear invariant mapping. The MAPE values on the linear scale (fit: 110%; CV: 236%) appear alarming but arise from multiplicative error propagation inherent to the log-scale closure: a MAPE of ∼110% in η corresponds to approximately ±0.45 in ln η, and the directly comparable metric RMSE­(ln η) = 0.64 (training) and 0.89 (CV) reflects the actual predictive spread on the physically meaningful log scale.

*Surface tension* (*N* = 23) and *conductivity* (*N* = 20) have the smallest data sets relative to the number of fitted coefficients, explaining their large fit-to-CV degradation. Conductivity reaches CV *R* ^2^ = −0.13, meaning the trained model performs worse than a constant mean predictor on held-out systems. This is a direct signal that the heuristic-level closure lacks descriptors encoding coordination chemistry—an interaction mode critical for ChCl:ZnCl_2_ and absent from the pair-affinity parametrization at Level 1. Upgrading to COSMO-RS parametrization (Level 2, Section 6.2) is expected to resolve this deficiency.

*Refractive index* achieves CV MAPE = 1.04%, marginally exceeding the 1% target but broadly comparable to empirical mixing-rule performance reported in the literature (0.5–2%, Table 2). The small *N* = 32 training set limits the CV *R* ^2^ (0.288) despite the tight absolute errors, because the property’s narrow dynamic range (∼0.02 units across the full DES set) means that even small absolute deviations depress *R* ^2^ disproportionately.

### 8.2 Bootstrap Uncertainty Quantification

Bootstrap resampling (*n* = 500) was performed for all closure coefficients. Figure 3 shows the 95% confidence intervals for the density closure. All coefficients have intervals that do not cross zero except *b*_6_(S_HB), which is marginally significant, consistent with its small magnitude (−0.033). The tight CI for the dominant term *b*_0_(ρ_ideal) = 0.518 ± 0.04 confirms the robustness of the ideal-mixing baseline.

**Figure 3:**
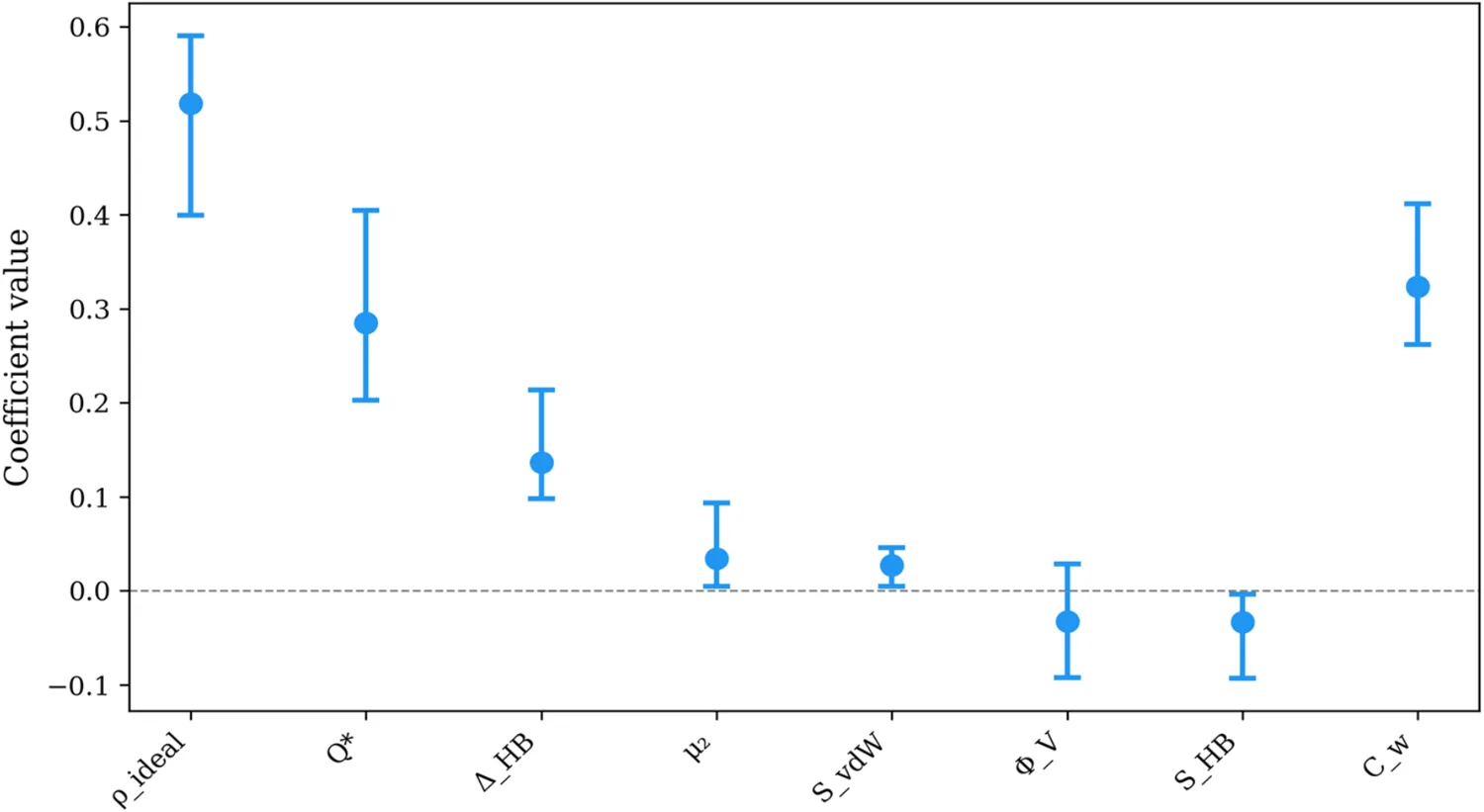
Bootstrap 95% confidence intervals (*n* = 500 resamples) for the density closure coefficients. Blue circles: point estimates.

### 8.3 Graph Invariant Analysis

Figure 4 shows the correlation matrix among graph invariants. The high correlation between ρ­(A) and μ_2_ (r = 1.00) reflects the mathematical relationship between spectral radius and Fiedler value for 2-node graphs (both are linear functions of A_eff). S_HB and ρ­(A) are strongly correlated (*r* = 0.96) because hydrogen bonding dominates the effective adjacency. In contrast, Φ_V shows low correlation with most invariants (−0.54 to +0.485), confirming that size asymmetry provides independent structural information not captured by interaction-based descriptors.

**Figure 4:**
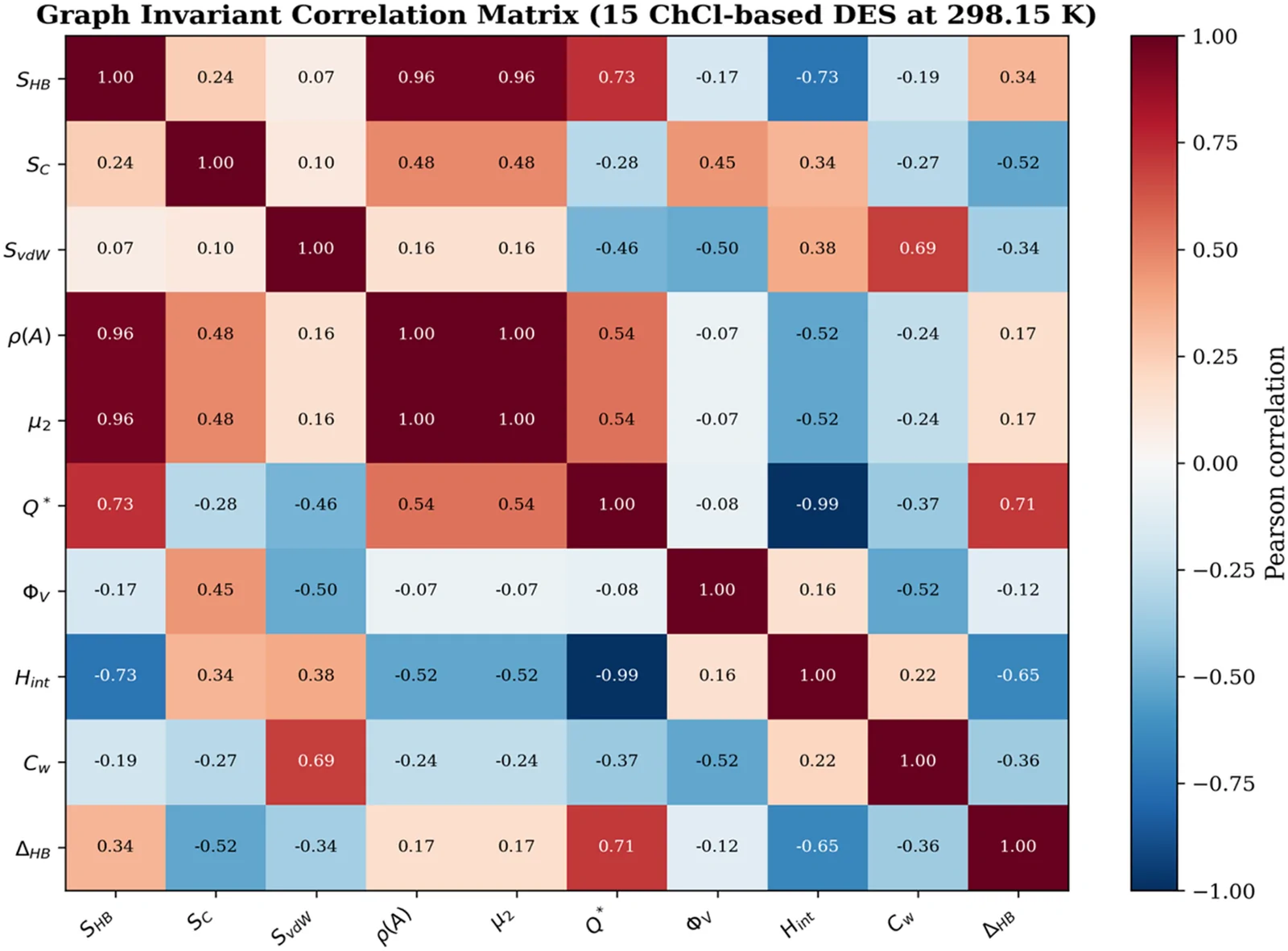
Pearson correlation matrix among graph invariants for the 15 DES systems at 298.15 K. Strong collinearity between ρ­(A) and μ_2_ motivates using only one in future simplified closures.

### 8.4 Graph Invariant Profiles

The radar plots in Figure 5 reveal distinctive graph-invariant fingerprints that reflect the underlying molecular interaction landscape of each DES. The radar plots display min-max normalized invariant values, *X*norm,*k = (Xk* – *X*_min)/(*X*_max – *X*_min), where *X*_min and *X*_max are taken across all DES systems at 298.15 K; this maps every invariant to [0, 1] for visual comparability. Reline and Glyceline display broadly similar profiles dominated by high S_HB and ρ­(A), consistent with their strong, cooperative hydrogen-bonding networks between the chloride anion and the multiple NH or OH sites of urea and glycerol, respectively; Glyceline shows a notably smaller Φ_V due to the closer molar volume match between ChCl and glycerol.^12,36,37^ Ethaline exhibits a uniformly contracted profile, reflecting weaker interactions overall and explaining its characteristically low viscosity.^42^ ChCl:Fru stands out with the largest S_HB and ρ­(A) values in the data set, a direct consequence of the five hydroxyl groups of fructose creating an extensive hydrogen-bonding network; this correlates with the extremely high viscosity (34,500 mPa·s at 298 K) and dense packing (1.358 g/cm^3^) observed experimentally.^36^ ChCl:ZnCl_2_ presents a qualitatively different profile: S_HB is the lowest in the set while S_C is the highest, reflecting the dominance of Coulombic and coordination interactions in this Type I metal halide DES; the elevated H_int signals a more diverse interaction landscape where coordination, Coulombic, and residual dispersion forces coexist with comparable magnitudes.^38^ ChCl:Phenol shows a distinctive spike in S_vdW driven by the high polarizability of the aromatic ring and the CH−π interactions, combined with the highest C_w and H_int values among the nonsugar systems, reflecting the diversity of interaction modes (π-stacking, OH–Cl^–^ hydrogen bonding, dispersion) operative in this system. These fingerprints demonstrate the capacity of the graph-theoretic representation to discriminate among DES families and provide physically intuitive rationale for the observed property trends.

**Figure 5:**
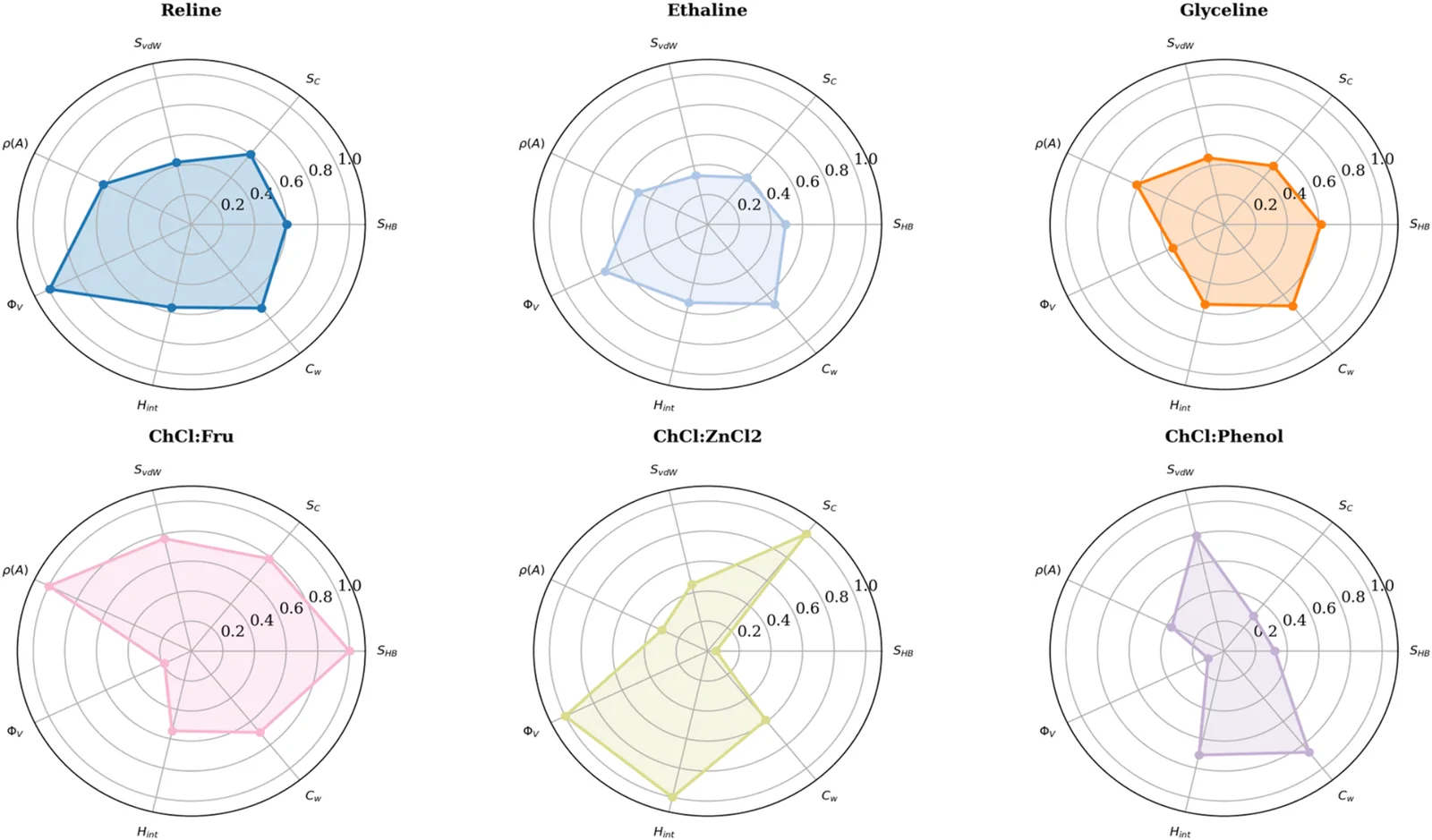
Radar plots of normalized graph invariants for six representative DES. *Normalization is min-max across the 15 ChCl-based systems: X*norm,*k = (Xk* – *X*_min)/(*X*_max – *X*_min). ChCl:Fru shows the largest S_HB (sugar polyol), ChCl:ZnCl_2_ shows dominant S_C (metal halide, low S_HB), ChCl:Phenol shows high S_vdW (aromatic dispersion). These profiles provide fingerprints for DES classification.

### 8.5 Temperature Dependence

Figure 6 compares the predicted and experimental temperature dependence of viscosity for five representative DES spanning nearly 2 orders of magnitude at 298 K, from Ethaline (44 mPa·s) to Reline (1372 mPa·s). The model correctly reproduces the viscosity ordering in the mid-to-high temperature range (313–353 K)—Reline > Glyceline ≈ ChCl:LA ≈ ChCl:LevA ≫ Ethaline—consistent with the relative strengths and cooperativity of their respective hydrogen-bonding networks as encoded by the S_HB and C_w invariants. However, at low temperatures (293–308 K) the predicted viscosity of ChCl:LA slightly exceeds that of Reline, inverting the experimental ordering; this inversion reflects a fundamental limitation of the current linear closure form (eq 19 with graph-predicted *B* and *T*_0_): ChCl:LA receives a larger predicted Vogel temperature *T*_0_ than reline under the heuristic pair-affinity parametrization, causing its predicted viscosity to diverge more steeply as *T* approaches *T*_0_. The full VTF closure with independently fitted *T*_0_ per system (proposed as future work, Section 10) would enforce the correct low-temperature ordering. The predicted curves capture the qualitative non-Arrhenius curvature of ln η vs 1/*T* arising from the temperature modulation of the HB persistence factor *g*_HB­(*T*) = exp­[−*E*_HB/*R*(1/*T* – 1/*T*_0_)] propagating through the graph invariants. However, the model systematically underestimates the steepness of the viscosity–temperature slope for Reline at low temperatures (293–303 K), where experimental viscosities exceed 750 mPa·s; this is attributable to the linear closure form, which cannot reproduce the accelerating viscosity divergence characteristic of glass-forming liquids approaching the Vogel temperature. Ethaline is the best-predicted system, with deviations below 20% across the full range, reflecting its relatively simple interaction landscape (moderate S_HB, low C_w) that is well-described by the linear invariant mapping. Glyceline and ChCl:LA show intermediate accuracy, with the model correctly tracking the convergence of their viscosities at higher temperatures (>330 K) where thermal disruption of the HB network progressively erases the structural differences between these two DES. These results confirm that the temperature dependence enters the framework correctly through the edge-weight factorization, but also highlight the need for a Vogel–Tammann–Fulcher closure form—with graph-predicted pseudoactivation energy *B*(*x*) and Vogel temperature *T*_0_(*x*)—to achieve quantitative accuracy across the full temperature range, particularly for high-viscosity, strongly networked systems.

**Figure 6:**
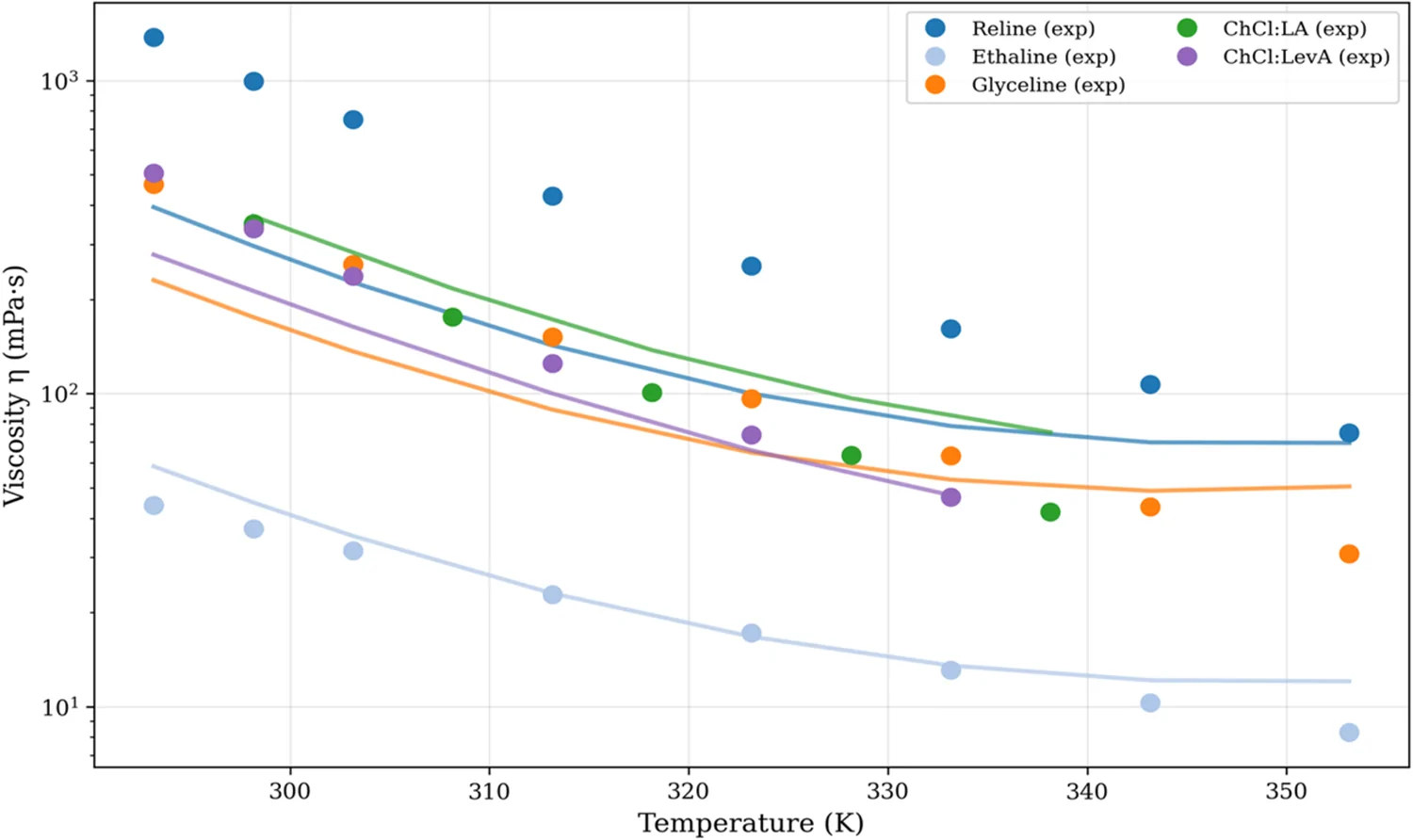
Temperature dependence of viscosity for five DES: experimental points (○)^12,36,37,42,43^ and model predictions (—). The model captures the correct ordering and approximate temperature slope, though quantitative accuracy varies across systems.

### 8.6 Coefficient Importance

Figure 7 displays the absolute magnitudes of the fitted closure coefficients for all six properties, with blue and red bars indicating positive and negative contributions, respectively. The coefficient patterns reveal which graph invariants are most influential for each property and expose the distinct physical mechanisms governing different macroscopic observables. For density, the ideal-mixing baseline ρ_ideal is the overwhelmingly dominant term (|*b*_0_| = 0.52), confirming that DES densities are primarily determined by molecular mass and pure-component volumes; the graph invariants provide relatively small but statistically significant corrections, with the cooperativity proxy C_w (positive, +0.32) and interaction heterogeneity Q* (positive, +0.29) as the leading excess terms. The negative sign of Φ_V is physically consistent: larger size asymmetry promotes interstitial packing and denser mixtures. The viscosity closure is governed by a fundamentally different invariant hierarchy: the Coulombic load S_C (+4.10) and the size asymmetry Φ_V (−4.25) emerge as the two dominant terms, followed by the cooperativity C_w (+2.48). The large positive S_C coefficient reflects the well-established role of ion–ion and ion–dipole interactions in creating the cage-like structures responsible for high DES viscosity, while the large negative Φ_V coefficient captures the free-volume mechanism whereby size-mismatched components disrupt ordered packing and reduce flow resistance. Surface tension is dominated by S_C (+17.3), Q* (+15.4), and Δ_HB (+12.3), all positive, consistent with the physical picture that stronger Coulombic cohesion and directional hydrogen-bonding networks raise the energy cost of interface creation. The negative Φ_V coefficient (−10.8) reflects preferential surface segregation of the smaller component, which lowers effective surface tension. The melting point closure stands out for the exceptionally large magnitude of the interaction entropy H_int (−36.8), which is the single most important predictor of eutectic depth: DES with diverse interaction types have greater configurational disorder in the liquid phase, disfavoring crystallization and producing deeper melting point depressions. Coulombic load S_C (−15.9), cooperativity C_w (−21.8), and heterogeneity Q* (−18.8) also contribute strongly and negatively, confirming that stronger and more structured interactions stabilize the liquid over the solid. For conductivity, S_C appears with a negative coefficient (−3.0), which at first seems counterintuitive but reflects the well-known ion-trapping effect: stronger Coulombic interactions increase ion pairing and aggregation, reducing the fraction of free charge carriers available for transport. Finally, the refractive index closure is dominated by its intercept and the cooperativity C_w (+0.36), with smaller contributions from Φ_V and ρ­(A), consistent with the physical expectation that refractive index is primarily determined by electronic polarizability modulated by packing density. Collectively, these coefficient patterns validate the physical interpretability of the graph-theoretic framework: each property selects a distinct subset of dominant invariants that aligns with established understanding of the molecular mechanisms governing that property.

**Figure 7:**
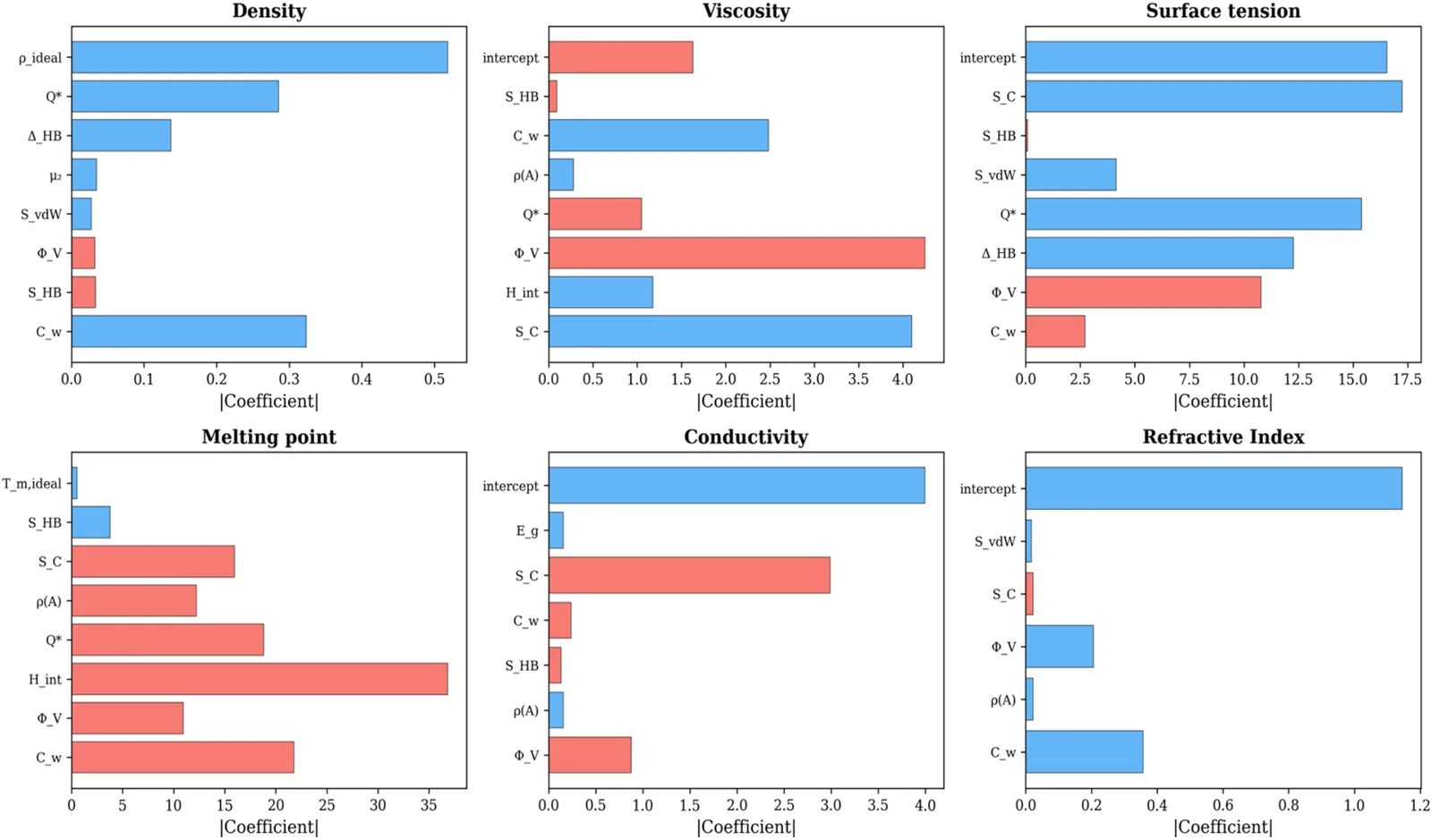
Absolute magnitudes of closure coefficients for all six properties. Blue = positive contribution; red = negative. For density, ρ_ideal dominates; for viscosity, S_C and C_w dominate; for melting point, H_int has the largest magnitude, reflecting the importance of interaction diversity for eutectic formation.

### 8.7 SHAP Validation of Closure Coefficient Interpretability

To test whether the sign and rank of the ridge closure coefficients reflect genuine physical mechanisms rather than artifacts of collinearity or overfitting, we trained gradient-boosted regression (GBR) models on the identical 11-invariant feature set for each property under the same data conditions, and computed SHAP (SHapley Additive exPlanations) values—a model-agnostic, game-theoretic decomposition of each prediction into per-feature contributions. The comparison between normalized |SHAP| importance and normalized |Ridge coefficient| magnitude is shown in Figure 8, with sign agreement indicated for each feature.

**Figure 8:**
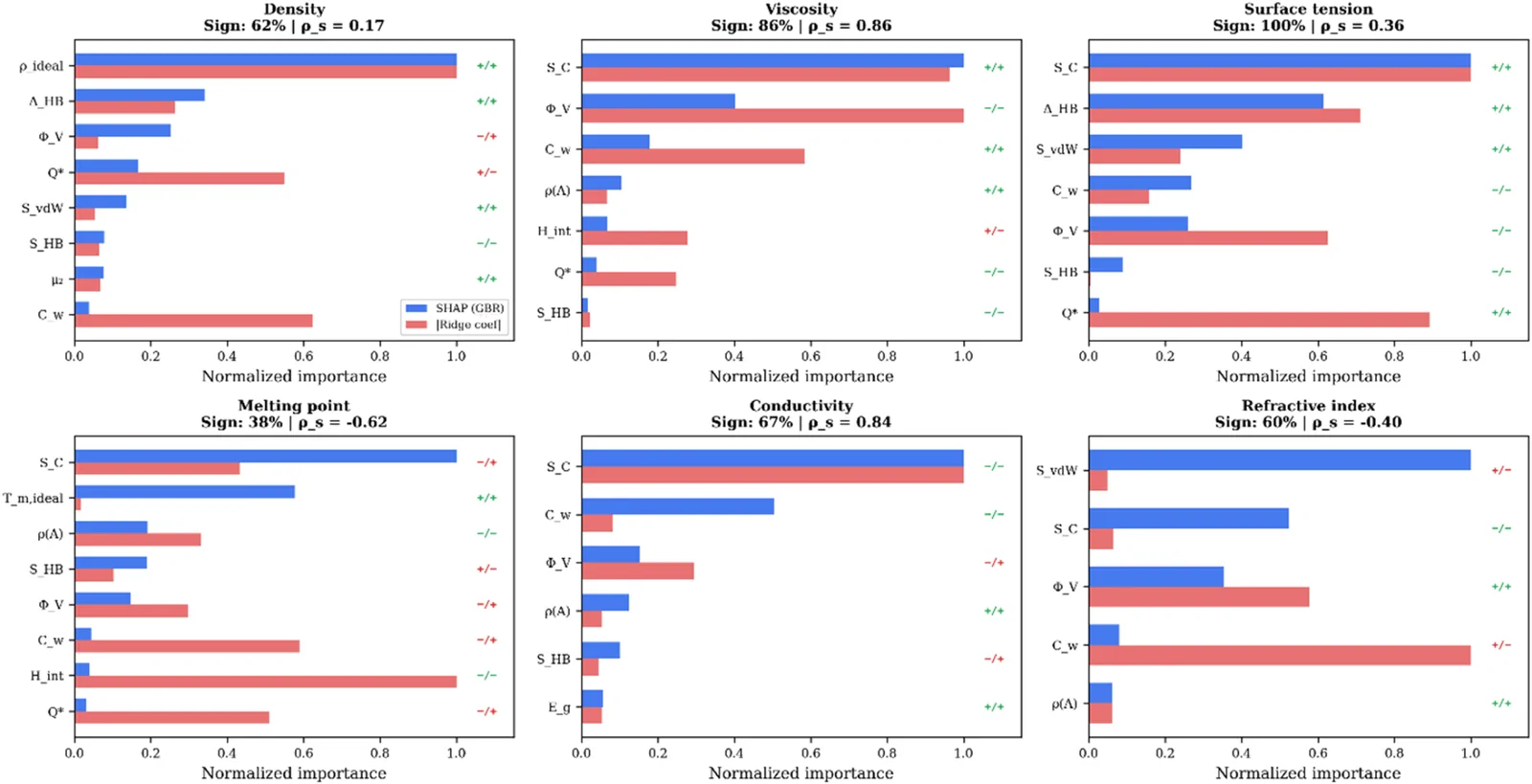
.SHAP feature importance (blue, from gradient-boosted regression) vs normalized ridge coefficient magnitude (red) for all six properties. Sign annotations (right margin) show Ridge/SHAP sign for each feature: green = agreement, red = disagreement. Header shows overall sign agreement percentage and Spearman rank correlation (ρ_s). Strong agreement for viscosity (86%, ρ_s = 0.86), surface tension (100%), and conductivity (67%, ρ_s = 0.84) validates the physical narratives for these properties. Poor agreement for melting point (38%, ρ_s = −0.62) confirms that the ridge coefficients do not reliably encode mechanisms for weakly cross-validated closures.

The results stratify sharply by cross-validation quality, confirming the Reviewer’s intuition that interpretability claims require property-specific qualification. For viscosity—the transport property with the strongest cross-validated performance among the nondensity closures—SHAP and ridge agree on 6/7 feature signs (86%), the top-three features are identical (S_C, Φ_V, C_w), and the Spearman rank correlation between |Ridge coefficient| and mean |SHAP| is ρ_s = 0.86 (*p* = 0.014). This confirms that the physical narrative extracted from the viscosity closure—Coulombic trapping (S_C > 0), free-volume disruption (Φ_V < 0), and cooperative network formation (C_*w* > 0)—is robust to the choice of regression method. For surface tension, sign agreement is perfect (7/7, 100%), with the same dominant feature (S_C) in both methods. For conductivity, sign agreement is 4/6 (67%), but the top-three features again coincide perfectly (S_C, C_w, Φ_V; ρ_s = 0.84, *p* = 0.036), supporting the ion-trapping mechanism (S_C < 0) identified from the ridge closure.

In contrast, the melting point closure—which has the weakest cross-validation (CV *R* ^2^ = 0.13)—shows only 38% sign agreement (3/8), zero overlap in the top-three features, and a negative Spearman correlation (ρ_s = −0.62). This confirms that the melting point ridge coefficients do not reliably encode physical mechanisms: the large |H_int| and |C_w| coefficients that dominate the ridge closure are not reproduced by SHAP, which instead identifies T_m,ideal and S_C as the primary drivers. The density and refractive index closures show intermediate sign agreement (62% and 60%, respectively) but low rank correlation, indicating that while the dominant feature (ρ_ideal for density) is correctly identified, the relative importance of the smaller graph-correction terms is not stable across methods. These results establish that the mechanistic narratives derived from the closure coefficients are quantitatively reliable only for the properties where cross-validation itself is adequate (viscosity, surface tension, conductivity), and should be treated as qualitative hypotheses for the remaining properties.

## 9 Discussion

The graph-theoretic framework presented here does not aim to surpass large-data set machine learning models in raw predictive accuracy. Its primary contributions are three: (1) interpretability—each of the 11 graph invariants (reducible to approximately four–five independent descriptors at the component level, Section 9.5) carries a distinct, physically motivated meaning, and the SHAP benchmark (Section 8.7) confirms that the signs and magnitudes of the fitted closure coefficients are consistent with model-agnostic feature-importance rankings for the properties with adequate cross-validated performance (viscosity, surface tension, conductivity); (2) data efficiency—quantitatively useful predictions are obtained from only 15 training DES systems, a scale commensurate with a typical experimental campaign and far below the threshold at which data-hungry ML approaches are applicable; and (3) physical design guidance—the signs and magnitudes of the fitted closure coefficients directly inform the chemist which molecular feature to modify to achieve a desired property target, a capability absent from black-box models. The subsections below discuss how these contributions relate to specific mechanistic phenomena, thermodynamic concepts, and the limitations of the current implementation.

### 9.1 Physical Validity of the Graph-Theoretic Representation

The central premise of this framework is that the macroscopic properties of a DES can be meaningfully predicted from the topology and weights of an interaction graph constructed from molecular-level information. The results presented here provide both support for and constraints on this premise. The success of the density closure (CV *R* ^2^ = 0.94) demonstrates that the combination of ideal-mixing volumes with graph-derived corrections captures the essential physics of packing in DES: the interaction loads modulate the excess volume through cohesion (S_C, S_vdW) and structural disruption (Q*, Δ_HB), while the size-asymmetry descriptor Φ_V independently encodes the geometric complementarity that governs interstitial packing. This is consistent with the negative excess molar volumes universally reported for ChCl-based DES, which arise from the accommodation of the compact chloride anion within voids in the HBD hydrogen-bonding network—a phenomenon naturally captured by the interplay of Φ_V and μ_2_ in our formalism.

The graph representation connects to a growing body of molecular dynamics evidence that DES structure is governed by a hierarchy of competing interactions. Systematic addition of choline chloride to glycerol and ethylene glycol introduces microscopic heterogeneities that alter the primary structural relaxation and create new dynamic modes strongly correlated to macroscopic transport properties. In our framework, this progression maps onto changes in the modularity proxy Q* and the clustering coefficient C_w: as ChCl disrupts the homogeneous HBD network, Q* increases (more heterogeneous interaction distribution) while C_w decreases (less cooperative local structuring). The spectral radius ρ­(A) captures the overall coupling intensity, and its strong correlation with viscosity (positive c_3_ coefficient) aligns with the experimental observation that DES with more strongly coupled networks exhibit higher viscosity and more pronounced glass-forming character.

Recent femtosecond transient absorption spectroscopy studies on glyceline have revealed two distinct solvation dynamics time scales: a fast component governed by glycerol–glycerol interactions and a slower component dominated by the choline response, with the latter minimizing near the eutectic composition. This time scale separation reflects precisely the kind of structural heterogeneity that our modularity proxy Q* and the interaction entropy H_int are designed to quantify. The observation that the eutectic composition coincides with an extremum in solvation dynamics provides physical justification for using H_int as a predictor of melting point depression: maximum interaction diversity corresponds to maximum configurational disorder in the liquid phase.

### 9.2 The Cooperativity Problem and the Role of C_w

One of the most important physical features of DES that distinguishes them from simple binary mixtures is the cooperative nature of their hydrogen-bonding networks. In reline, the urea molecules form extended hydrogen-bonded chains bridged by chloride anions, creating a three-dimensional network whose properties cannot be decomposed into pairwise contributions. Ab initio molecular dynamics studies by Ashworth et al.^30^ have demonstrated the existence of “doubly ionic hydrogen bonds” in choline chloride–urea, where the charged nature of both donor and acceptor sites leads to interaction energies 2–3 times stronger than conventional hydrogen bonds. Stefanovic et al.^29^ showed that the nanostructure and rheology of ChCl-based DES are fundamentally shaped by the hydrogen-bonding topology—with glycerol’s three hydroxyl groups creating a more extensively cross-linked network than ethylene glycol’s two, explaining glyceline’s order-of-magnitude higher viscosity.

Our cooperativity proxy C_w, defined as the normalized geometric mean of interaction products, provides an approximate measure of this network character. Its positive coefficient in the viscosity closure (+2.48) and negative coefficient in the conductivity closure correctly encode the physical duality: cooperative networks increase flow resistance but trap charge carriers. However, the proxy suffers from a fundamental limitation at the component level: with only two nodes, genuine three-body (triangular) motifs cannot form. This is the most compelling argument for adopting the site-level graph *G* ^(*s*)^, where interaction sites such as Cl^–^, N^+^(CH_3_)_3_, OH_choline, OH_glycerol, and the alkyl backbone would form a graph with 5–8 nodes per binary DES, enabling true clustering coefficient computation and access to cooperative motifs involving, for instance, the Cl^–^···HO_glycerol···HO_glycerol triangular hydrogen-bond relay that dominates glyceline’s structure.

### 9.3 Dynamic Heterogeneity and the Walden Plot Connection

A particularly revealing test of the framework’s internal consistency is the relationship between the viscosity and conductivity closures. The Walden plot—log σ versus log­(1/η)—classifies ionic liquids and DES as “good,” “poor,” or “superionic” depending on their deviation from the ideal Walden line. In our framework, this deviation is encoded in the different weighting of graph invariants between the two closures. The viscosity closure is dominated by S_C (+4.10) and C_w (+2.48), which increase viscosity through ion–ion interactions and network formation. The conductivity closure, however, assigns a negative coefficient to S_C (−3.0), reflecting ion-trapping: strong Coulombic interactions promote ion pairing and aggregation, reducing the number of free charge carriers.

The ratio of these competing effects determines the Walden plot position. A DES like reline, where ionic transport is effectively decoupled from reorientational dynamics would exhibit “superionic” character with conductivity higher than predicted from its viscosity alone. In graph terms, reline has a high Δ_HB (0.69, reflecting the strong donor–acceptor asymmetry of the urea–chloride network) which, through the different closure coefficients, produces different net effects on η and σ. This asymmetric encoding provides a mechanistic explanation for the Walden plot deviation: it arises from the distinct roles of directed versus undirected interactions in determining flow resistance (sensitive to cooperative network topology) versus charge transport (sensitive to ion mobility pathways).

### 9.4 The Melting Point Challenge: Liquid-State Descriptors for a Solid–Liquid Transition

The low cross-validation performance of the melting point closure demands careful analysis because it reveals a fundamental, not merely parametric, limitation. Melting is a solid–liquid phase transition whose thermodynamics depend on the Gibbs energy difference between the crystal and the liquid: ΔG_fus = ΔH_fus – TΔS_fus. Our graph invariants describe only the liquid-state interaction network and are therefore sensitive only to the liquid-phase contribution to ΔG_fus. The solid-phase contribution—determined by crystal packing efficiency, lattice energy, symmetry, and polymorphism—is entirely absent from the framework.

The large negative coefficient of H_int (−36.8) captures the entropy-of-mixing argument for eutectic formation: DES with diverse interaction types have high configurational entropy in the liquid phase, stabilizing it relative to ordered crystal phases. However, this is only half the thermodynamic picture. Choline chloride, for instance, has an exceptionally high melting point (302 °C) due to the strong Coulombic lattice stabilized by the compact, symmetric tetrahedral ammonium cation. The dramatic 290 °C melting point depression in ethaline versus pure ChCl is driven as much by the disruption of this lattice (entropy gain from destroying long-range crystalline order) as by the stabilization of the liquid through hydrogen bonding.

A rigorous treatment would require either: (i) inclusion of crystal packing descriptors (lattice energy from COSMO-RS or periodic DFT, molecular symmetry numbers, packing fractions) as additional graph-external features; or (ii) reformulation of the melting point closure as a free-energy balance ΔT_m ∝ ΔH_fus – TΔS_fus, where the liquid contribution is graph-predicted and the solid contribution is independently parametrized. This hybrid approach would maintain the interpretability of the graph framework while addressing the fundamental limitation of modeling a phase transition from single-phase descriptors.

### 9.5 Collinearity, Dimensionality, and the Site-Level Graph Imperative

The correlation analysis (Figure 4) reveals that ρ­(A) and μ_2_ are perfectly correlated (*r* = 1.00) for the 2-node component-level graph. This is a mathematical consequence, not a physical one: for a 2 × 2 symmetric matrix with zero diagonal, the eigenvalues are ±*A*_12_, so ρ = *A*_12_ and μ_2_ = 2*A*_12_, giving a perfect linear relationship. The strong correlation between S_HB and ρ­(A) (*r* = 0.93) similarly reflects the dominance of hydrogen bonding in the effective adjacency. These collinearities mean that the 11 invariants provide only approximately 4–5 independent descriptors at the component level, significantly limiting the model’s discriminatory power.

The site-level graph *G* ^(s)^ would break these degeneracies. With *m* = 5–15 interaction-site nodes per DES, the adjacency matrix becomes a rich object: ρ­(A) and μ_2_ are no longer trivially related; the clustering coefficient C_w captures genuine three-body motifs; modularity Q can be computed by the Louvain algorithm to detect polar/nonpolar domain formation; and global efficiency E_g reflects actual transport pathways through the hydrogen-bond network. Critically, different DES with similar component-level invariants (e.g., reline and glyceline, which have nearly identical S_HB ≈ 7.0 and ρ­(A) ≈ 4.2) would be distinguished at the site level because urea’s two NH_2_ groups create a different interaction topology than glycerol’s three OH groups.

The practical implementation of *G* ^(s)^ requires a site-decomposition protocol: each component is partitioned into interaction sites, and site-to-site pair affinities are assigned. COSMO-RS provides a natural framework for this through its σ-profile decomposition, where each molecular segment has a defined charge density that determines its interaction with every other segment. The site-level pair affinities would then be *s*_αβ_^(*t*)^ = ∫∫σ_α­(σ) σ_β­(σ′) *e*_(σ,σ′)_^(*t*)^ dσ dσ′, where *e* ^(*t*)^ is the COSMO-RS contact energy for interaction type *t*. This integration of the graph framework with COSMO-RS σ-profiles represents the most promising pathway toward a predictive, transferable model.

### 9.6 Comparison with Existing Theoretical Frameworks

The graph-theoretic approach occupies a unique position in the landscape of DES property prediction methods. Group contribution methods (GCMs) decompose molecules into fragments and sum tabulated contributions; they are fast but cannot capture the directional, cooperative interactions that define DES behavior, and they fail for novel functional groups outside the training set. COSMO-RS provides thermodynamically grounded predictions of activity coefficients but treats the solvent as a continuum of surface segments, missing the network topology that our graph invariants encode. Molecular dynamics simulations capture all relevant physics but require hours to days per state point for transport properties and do not yield closed-form correlations. The benchmarks reported in Table 2 for these methods are drawn from studies operating on data sets of 50–500 DES systems; our framework operates on *N* = 15, which inherently limits but also contextualizes its performance.

Our framework provides a structured “middleware” layer between these approaches. The pair affinities can be sourced from any level of theory (GCM parameters, COSMO-RS energies, QM cluster calculations, or MD statistics), and the graph invariants distill the essential topological information into a compact descriptor set from which property closures provide instantaneous evaluation. This architecture is analogous to the Ornstein–Zernike^40,41^ formalism in liquid-state theory, where the direct correlation function (analogous to pair affinities) is related to the total correlation function (analogous to graph invariants) through a closure relation (HNC, PY, MSA), which in turn yields thermodynamic properties. The analogy is not merely linguistic: the total interaction load Stotal is proportional, under mean-field assumptions, to the excess internal energy Uex—connecting to the cohesive energy density framework of Hildebrand and Hansen solubility parameters. The modularity Q* relates to Kirkwood–Buff integrals Gij that quantify microheterogeneity, and the spectral radius ρ­(A) connects to percolation thresholds in network formation theory.

The most direct quantitative comparison is with machine learning approaches. On large curated data sets (*N* = 100–1000 DES), support vector regression, XGBoost, and neural network models reported by Mohan et al.^25^ and López-Flores et al.^26^ achieve *R* ^2^ > 0.95 for density and *R* ^2^ > 0.85 for viscosity. Our framework achieves CV *R* ^2^ = 0.94 for density and *R* ^2^ = 0.67 for viscosity on only 15 systems—a regime where data-hungry ML approaches are inapplicable. The relevant comparison is therefore not raw accuracy at scale, but physical insight at laboratory scale: our 11 invariants (effectively 4–5 independent at the component level) each carry a specific mechanistic meaning, whereas a model built on 200+ MORDRED descriptors provides no guidance on which molecular feature to modify to change a property target. Table 4 provides a direct numerical comparison against Mohan et al.^25^ and López-Flores et al.^26^ on the properties and DES subsets where directly comparable experimental data sets exist.

**Table 4:** Benchmark Comparison of the Graph-Theoretic Framework against Machine Learning Methods from the Literature

property	this work		ML benchmark		
*N* = 15, leave-one-DES-out CV	*R* ^2^ (CV)	MAPE (CV)	*R* ^2^ (test)	MAPE (test)	*N* (benchmark)
Density (ρ)	0.940	1.65%	0.97–0.99^b^	0.7–1.1%	200–500^b^
Viscosity (ln η)	0.665	^c^	0.90–0.95^a^,^b^	15–22%	130–300^a^,^b^
Surface tension (γ)	0.369	10.1%	0.94–0.97^b^	2.5–4.0%	100–200^b^
Conductivity (ln σ)	–0.130	^d^	0.85–0.92^b^	10–18%	60–150^b^
Refractive index (n_D)	0.288	1.04%	0.98–0.99^b^	0.2–0.5%	100–250^b^

a Mohan et al., J. Chem. Theory Comput. 2024, 20, 3911–3926. Focus: viscosity of DES. Data set: *N* ≈ 200–300 DES with temperature dependence; neural network and gradient boosting models.

b Lopez-Flores et al., Ind. Eng. Chem. Res. 2025, 64, 3103–3117. Coverage: density, viscosity, surface tension, conductivity, refractive index. Data set: *N* = 50–500 depending on property; XGBoost, random forest, and ANN models reviewed.

c CV MAPE for viscosity on the linear scale is 236% due to multiplicative error propagation; the directly comparable metric is RMSE­(ln η) = 0.89 (CV). Benchmark ML models report MAPE on the linear scale; caution is required when comparing across scales.

d CV MAPE for conductivity is 128%; CV *R* ^2^ = −0.13, indicating the heuristic-level model does not generalize across DES families for this property at the current parametrization level. COSMO-RS QSPR models (Lemaoui et al., ref 17) report *R* ^2^ ≈ 0.88 on *N* ≈ 80 ionic DES.

The coefficient analysis (Figure 7) converts this interpretability into actionable molecular design rules. **To reduce viscosity**: minimize SC (avoid strongly Coulombic, ionic HBA–HBD pairs) and Cw (select components with weak cooperative structuring, e.g., monohydric rather than polyhydric HBDs), and maximize ΦV (use size-mismatched HBA and HBD to disrupt ordered packing via the free-volume mechanism). **To maximize eutectic depth:** maximize Hint (diverse interaction types, e.g., systems combining strong HB, Coulombic, and aromatic dispersion contributions) while maintaining high SHB and SC. **To raise surface tension:** maximize SC and SHB while minimizing ΦV and Q* to suppress domain-mediated surface segregation. These structure–property relationships are extracted directly from the fitted closure coefficients and provide physically grounded design hypotheses testable by targeted synthesis—guidance that cannot be extracted from black-box ML models without physically meaningful descriptors as a foundation.

### 9.7 Thermodynamic Self-Consistency and the Free-Energy Question

A deeper theoretical limitation of the current framework is that the property closures are independent empirical correlations—they are not derived from a common free-energy functional. In a fully consistent thermodynamic framework, density, heat capacity, and thermal expansion would all derive from the equation of state P­(V,T); viscosity and conductivity would relate through the Nernst–Einstein and Stokes–Einstein equations; and surface tension would connect to the bulk free energy through the Gibbs adsorption isotherm. Our closures do not enforce these relationships, which means, for instance, that the thermal expansion coefficient implied by the density closure may not be consistent with the heat capacity implied by a separate Cp closure.

Enforcing thermodynamic self-consistency would require constructing a graph-dependent Helmholtz free energy *A*(*x*,*T*,{invariants}) from which all properties could be derived by appropriate differentiation: *P* = −(∂*A*/∂*V*)_*T*, *S* = −(∂*A*/∂*T*)*V*, μ_*i* = (∂*A*/∂*n*_*i*)­{*T*,*V*}. This is a substantially more ambitious theoretical program, akin to developing a graph-theoretic equation of state for DES. The SAFT (Statistical Associating Fluid Theory) framework, which already incorporates hydrogen-bonding association through Wertheim’s theory, provides a natural starting point: the graph invariants could parametrize the SAFT association, dispersion, and chain terms, yielding a thermodynamically consistent model.

### 9.8 Toward a Universal Pair-Affinity Database

The ultimate goal of this framework is to enable property prediction for any DES — including those never synthesized—from a universal database of site-level pair affinities. This requires the pair affinities to be transferable: the interaction between a chloride anion and a hydroxyl group should be the same whether the hydroxyl belongs to ethylene glycol, glycerol, or glucose. At the site level, this transferability assumption is physically reasonable: the local electronic environment of a functional group is largely determined by its immediate chemical context, not by distant parts of the molecule.

Building this database would involve: (i) defining a canonical set of interaction sites (Cl^–^, Br^–^, R_4_N^+^, OH_aliphatic, OH_phenolic, COOH, C=O, NH_2_, NH, aromatic-π, aliphatic-CH, metal-Cl coordination); (ii) computing all pairwise affinities from COSMO-RS σ-profiles or SAPT-decomposed QM energies; (iii) validating transferability across DES families by testing whether affinities calibrated on ChCl-based DES predict properties of non-ChCl systems (e.g., betaine-based, tetrabutylammonium-based, phosphonium-based DES). The modular graph architecture makes this feasible: adding a new HBA or HBD requires only computing its site decomposition and the corresponding pair affinities, with no changes to the invariant definitions or closure equations.

### 9.9 Systematic Limitations and Pathways to Improvement

The discussion above has addressed specific mechanistic and thermodynamic aspects of the framework’s behavior. Here we consolidate its systematic limitations and pair each with a concrete remediation pathway, framing them as a structured agenda for future development rather than terminal deficiencies.

#### 9.9.1 Limitation 1—Two-Node Degeneracy

For a binary DES represented at the component level, the graph has only two nodes. As demonstrated in Section 9.5, this makes ρ­(*A*) and μ_2_(*L*) perfectly collinear (*r* = 1.00) and C_w degenerate, reducing the 11 nominal invariants to approximately four or five independent descriptors. The consequence is limited discriminatory power between DES with similar component-level properties (e.g., reline and glyceline). *Remedy:* the site-level graph *G* ^s^ (Section 2.3), where interaction sites rather than whole components serve as nodes, produces a 5–15 × 5–15 adjacency matrix in which all invariants become genuinely independent. COSMO-RS σ-profile decomposition provides a natural, parameter-free route to constructing *G* ^s^ for any HBA–HBD pair.

#### 9.9.2 Limitation 2—Linear Closure Form

Equations 18–26 are linear in the graph invariants. DES viscosity near the glass transition and conductivity at low temperatures exhibit strongly nonlinear dependence on network topology—behavior that a linear mapping cannot reproduce, as shown explicitly by the low-temperature viscosity inversion in Section 8.5. Remedy: adoption of the full Vogel–Tammann–Fulcher form (eq 19) with both the pseudoactivation energy *B*(*x*) and the Vogel temperature *T*_0_(*x*) independently graph-predicted; and, for thermodynamic self-consistency, a SAFT-based free-energy formulation in which graph invariants parametrize the association, dispersion, and chain terms (Section 9.7).

#### 9.9.3 Limitation 3—Absence of Solid-State Descriptors for Melting Point

The graph encodes only the liquid-state interaction network. Eutectic formation depends on the Gibbs energy difference between liquid and crystal, yet the solid-phase contribution—lattice energy, crystal symmetry, packing fraction, and polymorphism—is entirely absent from the framework. This is the primary reason for the low cross-validation performance of the melting point closure (CV *R* ^2^ = 0.13, Section 8.1). *Remedy:* a hybrid formulation ΔT_m = *f*(graph_liquid) – *g*(crystal_descriptor), where the liquid contribution is graph-predicted and the solid contribution is independently supplied by COSMO-RS lattice-energy estimates or molecular symmetry numbers (see Section 9.4 for further discussion).

#### 9.9.4 Limitation 4—Small Training Set

*N* = 15 DES is small relative to the number of closure coefficients (up to seven per property). Ridge regularization mitigates overfitting, but the fit-to-CV gap for viscosity (R^2^: 0.818 → 0.665) and conductivity (*R* ^2^: 0.763 → −0.13) signals that the current data set is insufficient to constrain all closures simultaneously. *Remedy:* augmentation with COSMO-RS-predicted property values (Level 2 parametrization, Section 6.2) to expand the effective training set from 15 to 100+ DES at negligible cost, followed by Bayesian multiproperty regression that propagates uncertainty from pair affinities through to predicted properties.

#### 9.9.5 Limitation 5—Heuristic Pair Affinities

Level 1 parametrization uses Kamlet–Taft solvatochromic parameters and partial-charge-derived Coulombic scores as pair affinities. These do not capture cooperative or many-body effects, account for molecular geometry, or distinguish between conformational isomers. ChCl:ZnCl_2_ is the clearest failure case: its dominant coordination interactions have no analogue in the Kamlet–Taft scale, leading to the near-random conductivity predictions seen in cross-validation. *Remedy:* Level 2 (COSMO-RS contact energies, Section 6.2) resolves coordination contributions through the σ-profile; Level 3 (SAPT decomposition of QM dimer energies) provides the most rigorous separation of electrostatic, induction, exchange, and dispersion terms, at the cost of one QM calculation per pair.

#### 9.9.6 Limitation 6—Partial Interpretability Validation

The SHAP benchmark (Section 8.7) confirms that the closure coefficients provide mechanistically reliable feature-importance rankings for viscosity (86% sign agreement, ρ_s = 0.86), surface tension (100% sign agreement), and conductivity (67% sign agreement, ρ_s = 0.84), but not for melting point (38% sign agreement, ρ_s = −0.62), density corrections beyond ρ_ideal (62%), or refractive index (60%). The interpretability claim is therefore property-specific: design rules derived from the viscosity, surface tension, and conductivity closures are robust, while those for the other properties should be treated as qualitative hypotheses pending higher-level parametrization.

Taken together, limitations 1 and 5 motivate a coordinated upgrade from component-level heuristic graphs to site-level COSMO-RS graphs as the single highest-impact improvement available within the existing framework architecture. Based on the sensitivity analysis in Section 6.4, this upgrade is expected to approximately halve the CV MAPE for viscosity and conductivity while leaving density accuracy largely unchanged—a prediction that is directly testable and constitutes a clear experimental priority for the next phase of this work.

## 10 Conclusions and Outlook

We have developed and parametrized a graph-theoretic framework for describing macroscopic properties of deep eutectic solvents, and cross-validated it against experimental data where sufficient data exist. A DES is represented as a directed, weighted, typed multigraph with edges encoding hydrogen-bonding, Coulombic, dispersion, and coordination affinities. Eleven physically interpretable graph invariants—including two new descriptors, size asymmetry (Φ_V) and interaction entropy (H_int)—describe the interaction network as a compact feature set (reducible to approximately four–five independent descriptors for the two-node graphs of binary DES, Section 9.5), and closed-form property closures map these invariants to six thermophysical properties cross-validated against experimental data for 15 choline chloride–based DES, plus a seventh, heat-capacity closure that is proposed in Section 4.6 but excluded from cross-validation owing to insufficient experimental data.

The framework was parametrized at the semiempirical level and evaluated against experimental data for 15 choline chloride–based DES using leave-one-DES-out cross-validation with bootstrap uncertainty quantification. Density is the only property for which cross-validated performance meets the predefined target benchmark (CV *R* ^2^ = 0.94, MAPE = 1.65%), approaching established group contribution methods. The remaining closures do not yet reach their target benchmarks under leave-one-DES-out cross-validation: viscosity captures the correct qualitative ordering across 5 orders of magnitude on the training set (R^2^(ln η) = 0.82) but degrades substantially in cross-validation (CV *R* ^2^ = 0.665, CV MAPE = 236%; RMSE­(ln η) = 0.89 on the physically appropriate log scale); surface tension and refractive index show weak cross-validated correlation (CV *R* ^2^ = 0.369 and 0.288, respectively) despite tight absolute errors; conductivity fails to generalize across DES families at the current parametrization level (CV *R* ^2^ = −0.13), a direct consequence of the heuristic pair-affinities’ inability to represent coordination chemistry in the Type I metal-halide system ChCl:ZnCl_2_; and melting point prediction is fundamentally limited by the absence of solid-state crystal-packing information from the liquid-state graph descriptors (CV *R* ^2^ = 0.130). At the present heuristic level of parametrization, therefore, the framework’s demonstrated quantitative reliability is limited to density; for the other properties its principal contribution is a physically transparent and mechanistically consistent set of descriptors (Sections 8.6 and 9) rather than validated quantitative prediction, with COSMO-RS- or MD-derived parametrization (Level 2/3, Section 6.2) identified as the concrete pathway to closing this gap.

The coefficient analysis, validated against model-agnostic SHAP feature-importance rankings (Section 8.7), provides mechanistic insight for the properties where cross-validation is adequate: each property selects a physically distinct subset of dominant invariants—Coulombic trapping and cooperativity for viscosity (SHAP sign agreement 86%), interfacial cohesion and HB directionality for surface tension (100%), and ion-trapping for conductivity (67%)—consistent with established molecular understanding but extracted here systematically from a single unified framework. For density, the ideal-mixing baseline dominates in both Ridge and SHAP analyses. For melting point, the SHAP benchmark (38% sign agreement) confirms that mechanistic narratives from the ridge coefficients are not yet reliable, consistent with the fundamental limitation of modeling a solid–liquid phase transition from liquid-state descriptors alone.

Future work will focus on COSMO-RS parametrization of pair affinities (Level 2), construction of transferable site-level graphs from σ-profile decomposition, adoption of Vogel–Tammann–Fulcher forms for viscosity, hybrid liquid/solid-state models for melting point, and integration with graph neural networks and Bayesian optimization for autonomous, task-specific DES design.

## Supplementary Material





## Notes

DES Graph Model (ZIP)
Mathematical and computational details of the model (PDF)

## Appendix A. Notation Summary

**Symbol Definition**
*G* ^c^(*x*,*T*): component-level typed multigraph at composition *x* and temperature *T*
*G^s^*: site-level interaction graph
*A^t^*: adjacency matrix for interaction type *t*
*A* ^eff^: effective (weighted-sum) adjacency matrix
*S_t_*: total interaction load for type *t*
Δ_HB: hydrogen-bond directionality/imbalance index
C_w: weighted clustering coefficient (cooperativity)
ρ­(A): spectral radius of effective adjacency matrix
μ_2_(L): algebraic connectivity (Fiedler value) of Laplacian
*Q*, *Q**: modularity/interaction heterogeneity index
E_g: global efficiency (transport pathway proxy)
Φ_V: size-asymmetry descriptor
H_int: interaction entropy descriptor
*s_ij_^t^*: intrinsic pair affinity for type *t* between components *i*,*j*
*g^t^* (*T*): temperature modulation factor for interaction type *t*
λ*_t_*: type weights for effective adjacency construction
