# Supplementary material for: Graph Theory of Deep Eutectic Solvents: From Typed Interaction Networks to Property Prediction
Source: J Phys Chem B. 2026 Aug 28;130(36):9123–41. doi: 10.1021/acs.jpcb.6c01054 (PMC13573610; doi:10.1021/acs.jpcb.6c01054)
Supplement: Supplementary file 1 [file jp6c01054_si_002.pdf]

Graph Theory of Deep Eutectic Solvents: From Typed Interaction Networks to Property Prediction

Santiago Aparicio

Department of Chemistry, University of Burgos, 09001 Burgos, Spain

sapar@ubu.es

S1. Mathematical Details of Graph Invariants

S1.1 Perron–Frobenius theorem and spectral radius

For a non-negative, irreducible matrix A, the Perron–Frobenius[39] theorem guarantees that the spectral radius ρ(A) is a simple eigenvalue with a strictly positive eigenvector. In our context, the effective adjacency matrix A^{eff} is non-negative by construction (all edge weights are non-negative) and irreducible when all components interact with all others (a reasonable assumption for DES). The Perron eigenvector gives the "interaction importance" of each component: the component with the largest entry in the Perron eigenvector is the most strongly coupled to the network.

S1.2 Fiedler vector and spectral partitioning

The eigenvector corresponding to μ_2 (Fiedler vector) partitions the graph into two groups such that the edge weight cut between them is minimized (normalized by group sizes). For DES, the Fiedler vector reveals the natural division between HBA-like and HBD-like subcommunities. When μ_2 is close to zero, the system approaches phase separation; when μ_2 is large relative to the maximum eigenvalue, the system is well-mixed.

S1.3 Relationship between invariants and thermodynamic quantities

The total interaction load S_total = $\Sigma_i S_i$ is proportional (under the mean-field approximation) to the excess internal energy of mixing: $U^{ex} \approx -\alpha$ S_total, where α depends on the energy scale of the pair affinities. This provides a direct thermodynamic interpretation of the graph invariants and can serve as a calibration point for the overall energy scale.

The interaction entropy H_int is related to the excess configurational entropy of mixing. Mixtures with high H_int (diverse interaction types) have more ways to arrange their interaction

network, contributing positively to the entropy of mixing and stabilizing the liquid phase relative to ordered crystalline phases.

S2. Computational Implementation Notes

All graph invariants can be computed with standard linear algebra operations on matrices of dimension $n \times n$ (component level) or $m \times m$ (site level). For typical DES systems, $n = 2$–4 and $m = 5$–15, so all computations are trivial and exact (no iterative solvers needed). The eigenvalue problems for ρ and μ_2 can be solved analytically for $n = 2$–3 or with standard LAPACK routines for larger systems.
