# Supplementary material for: Graph Theory of Deep Eutectic Solvents: From Typed Interaction Networks to Property Prediction
Source: J Phys Chem B. 2026 Aug 28;130(36):9123–41. doi: 10.1021/acs.jpcb.6c01054 (PMC13573610; doi:10.1021/acs.jpcb.6c01054)
Supplement: Supplementary file 2 [file jp6c01054_si_001.zip › DES_Graph_Model_SI.html]

DES Graph Theory Property Predictor


# DES Graph Theory Property Predictor

Interactive typed multigraph framework for DES thermophysical property prediction

Supporting Information — Aparicio, S. J. Phys. Chem. B 2026

Input Parameters

DES System

— Select preset DES —
Reline (ChCl:Urea 1:2)
Ethaline (ChCl:EG 1:2)
Glyceline (ChCl:Glycerol 1:2)
ChCl:Lactic acid 1:2
ChCl:Malonic acid 1:1
ChCl:Oxalic acid 1:1
ChCl:Levulinic acid 1:2
ChCl:Phenol 1:2
ChCl:Acetic acid 1:2
ChCl:1,4-Butanediol 1:3
ChCl:Fructose 1:1
ChCl:Citric acid 1:1
ChCl:pTSA 1:1
ChCl:ZnCl₂ 1:2
ChCl:Phenylacetic acid 1:2
✦ Custom DES

Temperature (K)

#### HBD Descriptors

α (HB donor)

β (HB acceptor)

Polarizability

Charge density

n(HB sites)

Aromatic (0/1)

#### Component Properties

HBA:HBD ratio

M\_HBD (g/mol)

Vm\_HBD (cm³/mol)

Coordination

▶ Compute Properties
Compare All Preset DES

Closure Equations

ρ = 0.518·ρ\_id + 0.285·Q\* + 0.137·Δ\_HB + 0.035·μ₂ + 0.027·S\_vdW − 0.032·Φ\_V − 0.033·S\_HB + 0.324·C\_w

ln η = −1.63 − 0.094·S\_HB + 2.48·C\_w + 0.28·ρ(A) − 1.05·Q\* − 4.25·Φ\_V + 1.18·H\_int + 4.10·S\_C

γ = 16.6 + 17.3·S\_C − 0.08·S\_HB + 4.15·S\_vdW + 15.4·Q\* + 12.3·Δ\_HB − 10.8·Φ\_V − 2.72·C\_w

T\_m = 0.558·T\_id + 3.79·S\_HB − 15.9·S\_C − 12.2·ρ(A) − 18.8·Q\* − 36.8·H\_int − 10.9·Φ\_V − 21.8·C\_w

Properties

Invariants

Radar

Comparison

Select a DES and click Compute Properties.

Run a prediction to see invariant values.

Click Compare All Preset DES.

Graph Theory of Deep Eutectic Solvents: From Typed Interaction Networks to Property Prediction  
S. Aparicio — University of Burgos — Supporting Information S1 — 2026
